# Supplementary material for: Development and validation of the pandemic fatigue scale
Source: Nat Commun. 2023 Oct 10;14:6352. doi: 10.1038/s41467-023-42063-2 (PMC10564944; doi:10.1038/s41467-023-42063-2)
Supplement: Supplementary file 1 — Supplementary Information [file 41467_2023_42063_MOESM1_ESM.pdf]

Development and Validation of the Pandemic Fatigue Scale

Supplementary Information

Lau Lilleholt[1,2,3], Ingo Zettler[1,2], Cornelia Betsch[4,5], and Robert Böhm[1,2,6]

[1]Department of Psychology, University of Copenhagen, Øster Farimagsgade 2A, 1353 Copenhagen, Denmark; [2]Copenhagen Center for Social Data Science (SODAS), University of Copenhagen, Øster Farimagsgade 5, 1353 Copenhagen, Denmark; [3]Centre for the Experimental-Philosophical Study of Discrimination, Aarhus University, Bartholins Allé 7, 8000 Aarhus, Denmark; [4]Institute for Planetary Health Behaviour, University of Erfurt, Nordhäuser Str. 63, 99089 Erfurt, Germany; [5]Health Communication, Bernhard-Nocht-Institute for Tropical Medicine, Bernhard-Nocht-Strasse 74, 20359 Hamburg, Germany; [6]Faculty of Psychology, University of Vienna, Universitätsstrasse 7, 1010 Vienna, Austria

[*]Correspondence to: llj@psy.ku.dk

Supplementary Note 1

Development of Public Adherence to Recommended Health-Protective Behaviors over Time

As observed elsewhere[1–3], we find a significant decrease in people's tendency to adhere to physical distancing measures ($\beta_{standardized}$ = -.32, t(15,983) = -40.84, $p_{two\text{-}tailed}$ < .001, Cohen's f^2_{model} = .104, , 95% CI [-.33, -.30]), uphold hygienic practices ($\beta_{standardized}$ = -.11, t(15,983) = -16.00, $p_{two\text{-}tailed}$ < .001, Cohen's f^2_{model} = .016, 95% CI [-.12, -.10]), wear masks ($\beta_{standardized}$ = -.11, t(15,983) = -10.85, $p_{two\text{-}tailed}$ < .001, Cohen's f^2_{model} = .007, 95% CI [-.13, -.09]), and keep themselves informed about the pandemic ($\beta_{standardized}$ = -.21, t(15,060) = -19.71, $p_{two\text{-}tailed}$ < .001, Cohen's f^2_{model} = .026, 95% CI [-.23, -.19]) from October 2020 to September 2021 in the Danish repeated cross-sectional survey (Figure S8). In line with the results from the Danish repeated cross-sectional survey, we further observe a decline in people's tendency to adhere to physical distancing measures ($\beta_{standardized}$ = -.11, t(14,553) = -18.54, $p_{two\text{-}tailed}$ < .001, Cohen's f^2_{model} = .024, 95% CI [-.13, -.10]), wear masks ($\beta_{standardized}$ = -.05, t(17,801) = -8.60, $p_{two\text{-}tailed}$ < .001, Cohen's f^2_{model} = .004, 95% CI [-.06, -.04]), and keep themselves informed about the pandemic ($\beta_{standardized}$ = -.11, t(17,944) = -10.17, $p_{two\text{-}tailed}$ < .001, Cohen's f^2_{model} = .006, 95% CI [-.14, -.09]) from October 2020 to September 2021 in the German repeated cross-sectional survey. Finally, in the Danish panel survey there was also a significant decline in people's tendency to adhere to physical distancing measures ($\beta_{standardized}$ = -.15, t(477.17) = -11.30, $p_{two\text{-}tailed}$ < .001, marginal/conditional Cohen's f^2_{model} = .036/1.252, 95% CI [-.18, -.12]), uphold hygienic practices ($\beta_{standardized}$ = -.11, t(454.42) = -9.92, $p_{two\text{-}tailed}$ < .001, marginal/conditional Cohen's f^2_{model} = .016/ 3.019, 95% CI [-.13, -.09]), and keep themselves informed about the pandemic ($\beta_{standardized}$ = -.18, t(400.40) = -10.95, $p_{two\text{-}tailed}$ < .001, marginal/conditional Cohen's f^2_{model} = .016/ 4.132, 95% CI [-.21, -.15]) from October 2020 to June 2021.

Supplementary Note 2

Exploratory Factor Analysis based on Polychoric Correlations

Acknowledging that treating ordinal data as continuous may introduce bias[4–6], we conducted another exploratory factor analysis based on polychoric correlations rather than Pearson product-moment correlations. As for the exploratory factor analysis based on Pearson product-moment correlations, we verified the sampling adequacy of the data using the Kaiser-Meyer-Olkin (KMO) test[7] and found it to be acceptable (overall KMO = .93; all KMO values for individual items are > .86). Bartlett's test of sphericity[8] further indicated that the item correlations were sufficiently large for conducting an exploratory factor analysis ($X^2(45) = 5442.45$, $p < .001$). To determine the number of factors to extract, we once again considered the scree test[9], Glorfeld's modified parallel analysis[10,11], the very simple structure criterion[12], and the Velicer's minimum average partial criterion[13], which combined indicated that either a one-, two-, or three-factors solution would best reflect the data (Figure S21 and Table S19).

Considering a one-, two-, and three-factor solution, the exploratory factor analysis revealed that a two-factor model fit the data better (RMSR = .02, RMSEA = .07, TLI = .97) than a one-factor model (RMSR = .07, RMSEA = .15; TLI = .83), as indicated by the RMSR being closer to zero[14], a difference in RMSEA > .015[15], and a TLI above .95[16]. A three-factor model also fit the data well (RMSR = .01, RMSEA = .04, TLI = .99), but not much better than the two-factor model. Thus, among the three models considered, a two-factor model is arguably superior as it strikes a better balance between model fit and model parsimony[14]. Of note, the two-factor model explained 60.99% of the variance, with the first factor (information fatigue) accounting for 21.00% of the variance and the second factor (behavioral fatigue) 39.99%.

Reducing the length of the behavioral fatigue subscale, we end up with the same three items as for the exploratory factor analysis based on Pearson product-moment correlations. The final two-factor model with three items per factor fit the data well (RMSR = .01, RMSEA = .04, TLI = .99), and explained 64.48% of the variance, with the information fatigue factor accounting for 33.22% of the variance and the behavioral fatigue factor 31.26%. Standardized factor loadings, communalities, uniqueness, and complexity for the final two-factor model are presented in Table S20 together with the six items retained.

Supplementary Note 3

Confirmatory Factor Analysis using Robust Diagonally Weighted Least Squares Estimation

Accounting for the fact that treating ordinal data as continuous may lead to biased estimates even when using robust maximum likelihood estimation[17], we refitted all confirmatory factor analyses reported in the main manuscript while treating the data as ordinal using robust diagonally weighted least squares estimation[18]. Treating the data as ordinal we find that a two-factor model, as well as a second-order model with two subfactors, fit the data well in both Denmark (RMSEA = .09, SRMR = .03, TLI = .98, CFI = .99) and Germany (RMSEA = .08, SRMR = .02, TLI = .99, CFI = .99). Moreover, as for the confirmatory factor analysis based on robust maximum likelihood estimation, a one-factor model did not fit the data well in neither Denmark (RMSEA = .23, SRMR = .08, TLI = .88, CFI = .93) nor Germany (RMSEA = .19, SRMR = .06, TLI = .94, CFI = .96). The fully standardized factor loadings and (residual) variances for both the two-factor and second-order models fitted with robust diagonally weighted least squares estimation are shown in Figure S24.

Supplementary Note 4

Measurement Invariance Testing using Robust Diagonally Weighted Least Squares Estimation

Using robust diagonally weighted least squares estimation[18], we first tested for configural invariance by fitting a multi-group confirmatory factor analysis with no equality constraints across countries. In specifying the model, we relied on the identification strategy proposed by Yoon and Millsap (2007)[19] and further fixed all residual variances to one in order to freely estimate all thresholds[20]. The resulting model fit the data well suggesting that the PFS is configurally invariant across Denmark and Germany (RMSEA = .09 SRMR = .03, TLI = .99, CFI =.99). Next, we tested for metric invariance by constraining the factor loadings across countries to equality and comparing the fit of this constrained model to the fit of the first model with no equality constraints. Comparing the fit of these two models using Cheung and Rensvold's (2002) ΔCFI < -.01 criterion[21], we find the PFS to be metrically invariant across Denmark and Germany (ΔCFI = -.008). In light of these results, we thus turned to test for scalar invariance by additionally constraining the thresholds across countries to equality and comparing the fit of this model to the fit of the model in which only the factor loadings were constraint to equality. Considering the relative fit of these two models, we find the PFS to be scalarly non-invariant across Denmark and Germany (ΔCFI > -.01). As a final step, we therefore proceeded to test for partial scalar invariance by freeing the thresholds of the fourth item of the PFS (i.e., "I feel strained from following all of the behavioral regulations and recommendations around COVID-19"). Freeing the thresholds of the fourth item and comparing the fit of this model to the fit of the model in which only the factor loadings were constraint to equality, we find support for partial scalar invariance of the PFS (ΔCFI = -.010).

Supplementary Note 5

Confirmatory Factor Analysis based on Data from the U.S.

To confirm the factor structure of the PFS in the U.S. experimental sample, we conducted another confirmatory factor analysis. Using both robust maximum likelihood estimation (RMSEA = .08, SRMR = .04, TLI = .97, CFI = .98) and robust diagonally weighted least squares estimation (RMSEA = .10, SRMR = .03, TLI = .99, CFI = .99), we find that a two-factor model, as well as a second-order model with two subfactors, fit the data well. The fully standardized factor loadings and (residual) variances for both the two-factor and second-order models fitted with robust maximum likelihood estimation and robust diagonally weighted least squares estimation are presented in Figure S25.

Supplementary Note 6

Correlates of Pandemic Fatigue in the U.S.

Exploring the correlates of pandemic fatigue in the U.S. experimental sample we find no relation between people's age, gender, and education and their experience of pandemic fatigue (Figure S26). Conversely, in line with the results from the Danish and German repeated cross-sectional surveys, we find a negative link between people's cognitive risk perceptions regarding COVID-19 and their experience of pandemic fatigue ($\beta_{standardized}$ = -.15, t(1,552) = -4.22, $p_{two\text{-}tailed}$ < .001, Cohens $f^2_{predictor}$ = .011, 95% CI [-.22, -.08]).

Supplementary Note 7

Robustness of the Experimental Results

Exploring the robustness of the experimental results, we find the impact of our experimental manipulation of pandemic fatigue on people's intention to adhere to the four health-protective behaviors

of interest to be statistically significant, even after controlling for participants' age, gender, education, and cognitive risk perceptions regarding COVID-19. That is, controlling for these factors in an ordinary least square regression analysis, we still find that participants in the high pandemic fatigue condition expressed weaker intentions to adhere to the four health-protective behaviors in question as compared to participants in both the low pandemic fatigue condition ($\beta = .29$, $t(1,550) = 4.31$, $p_{\text{two-tailed Bonferroni-adjusted}} < .001$, 95% CI [.16, .43]) and the control condition ($\beta = .19$, $t(1,550) = 2.88$, $p_{\text{two-tailed Bonferroni-adjusted}} = .012$, 95% CI [.06, .33]). Moreover, across all three experimental conditions (Figure S22), we find a negative relation between pandemic fatigue and people's intention to adhere to physical distancing measures ($\beta_{\text{standardized}} = -.52$, $t(1,551) = -15.57$, $p_{\text{two-tailed}} < .001$, Cohens $f^2_{\text{predictor}} = .156$, 95% CI [-.59, -.46]), uphold hygienic practices ($\beta_{\text{standardized}} = -.29$, $t(1,551) = -9.21$, $p_{\text{two-tailed}} < .001$, Cohens $f^2_{\text{predictor}} = .055$, 95% CI [-.35, -.23]), wear masks ($\beta_{\text{standardized}} = -.40$, $t(1,551) = -11.00$, $p_{\text{two-tailed}} < .001$, Cohens $f^2_{\text{predictor}} = .078$, 95% CI [-.47, -.33]), and keep themselves informed about the pandemic ($\beta_{\text{standardized}} = -.75$, $t(1,551) = -19.92$, $p_{\text{two-tailed}} < .001$, Cohens $f^2_{\text{predictor}} = .256$, 95% CI [-.82, -.68]).

Figures and Tables

Figure S1. Development of pandemic fatigue over time controlling for time dependent contextual factors.

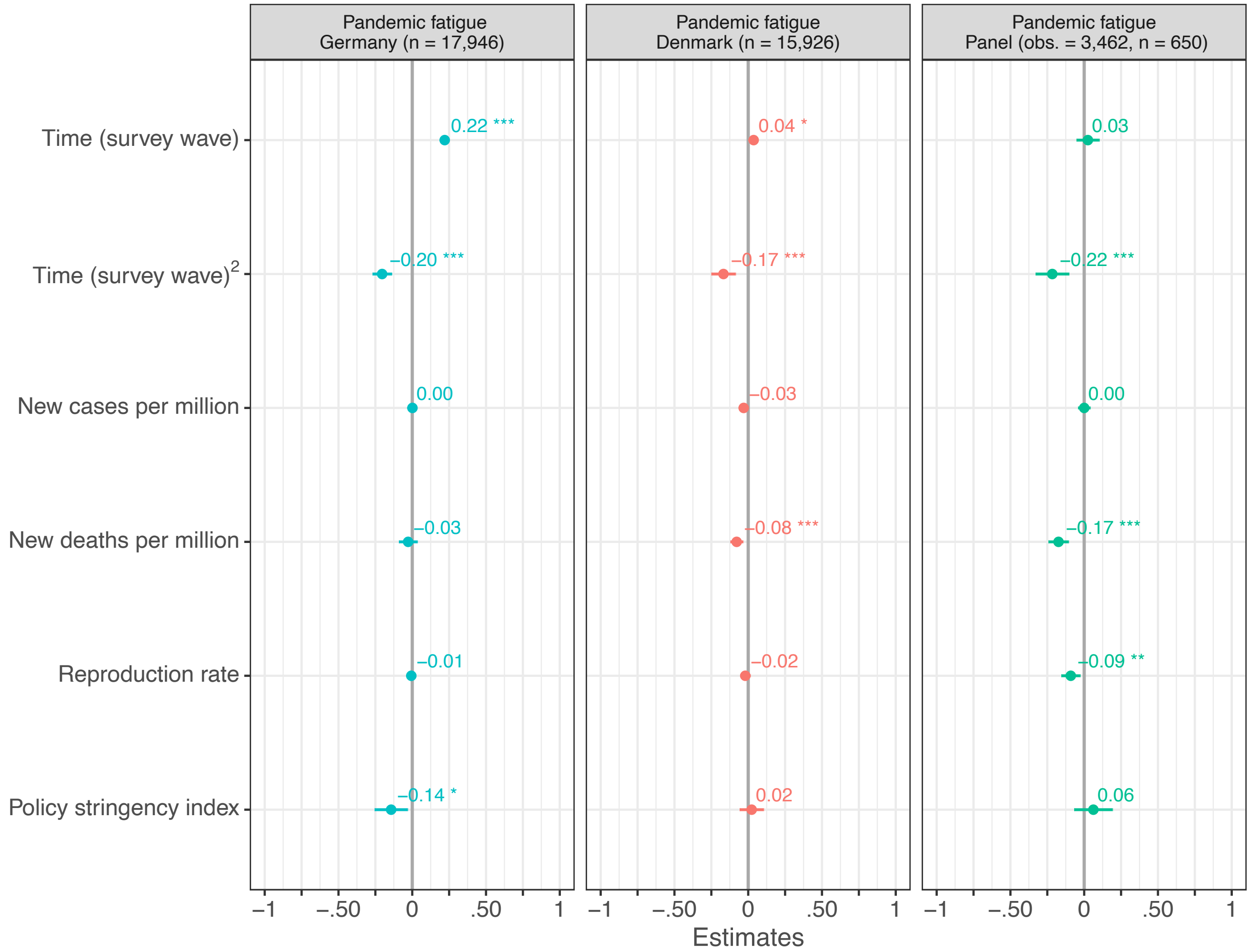

Note. Figure S1 shows standardized β coefficients with 95% confidence intervals based on ordinary least squares and mixed-models regressions with data from the Danish and German repeated cross-sectional surveys and the Danish panel survey. All continuous predictors have been mean-centered and scaled by 1 standard deviation. The p-values have not been adjusted for multiple comparisons and are presented as follows: $^{***}p_{\text{two-tailed}} < .001$; $^{**}p_{\text{two-tailed}} < 0.01$; $^{*}p_{\text{two-tailed}} < 0.05$. Exact p-values for all models are presented in the R-output which has been deposited on the Open Science Framework at: http://dx.doi.org/10.17605/OSF.IO/XD463.

Figure S2. Pairwise correlations for all variables considered in the Danish repeated cross-sectional survey.

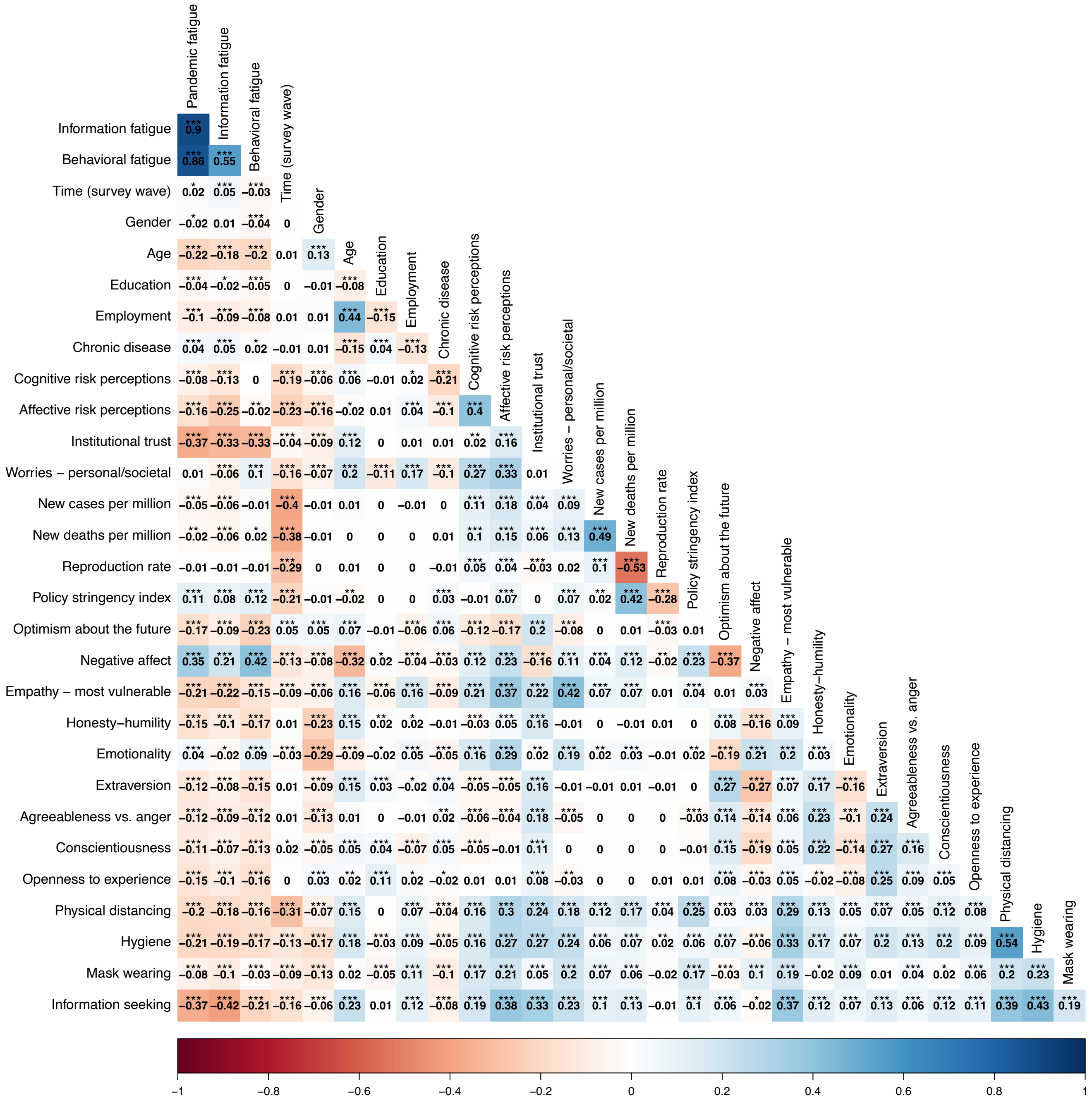

Note. For all correlations n ≤ 15,318 and n ≥ 14,388. Gender (male = 1, female = 0); Education (10 years or more = 1, less than 10 years = 0); Employment (unemployed = 1, employed = 0); Chronic disease (no = 1, yes = 0). The p-values have not been adjusted for multiple comparisons and are presented as follows: $^{***}p_{\text{two-tailed}} < .001$; $^{**}p_{\text{two-tailed}} < 0.01$; $^{*}p_{\text{two-tailed}} < 0.05$. The gender variable refers to participants self-identified gender as presented to them in the surveys. Participants who did not identify as either male or female are not included in the analyses due to an insufficient number of observations.

Figure S3. Pairwise correlations for all variables considered in the German repeated cross-sectional survey.

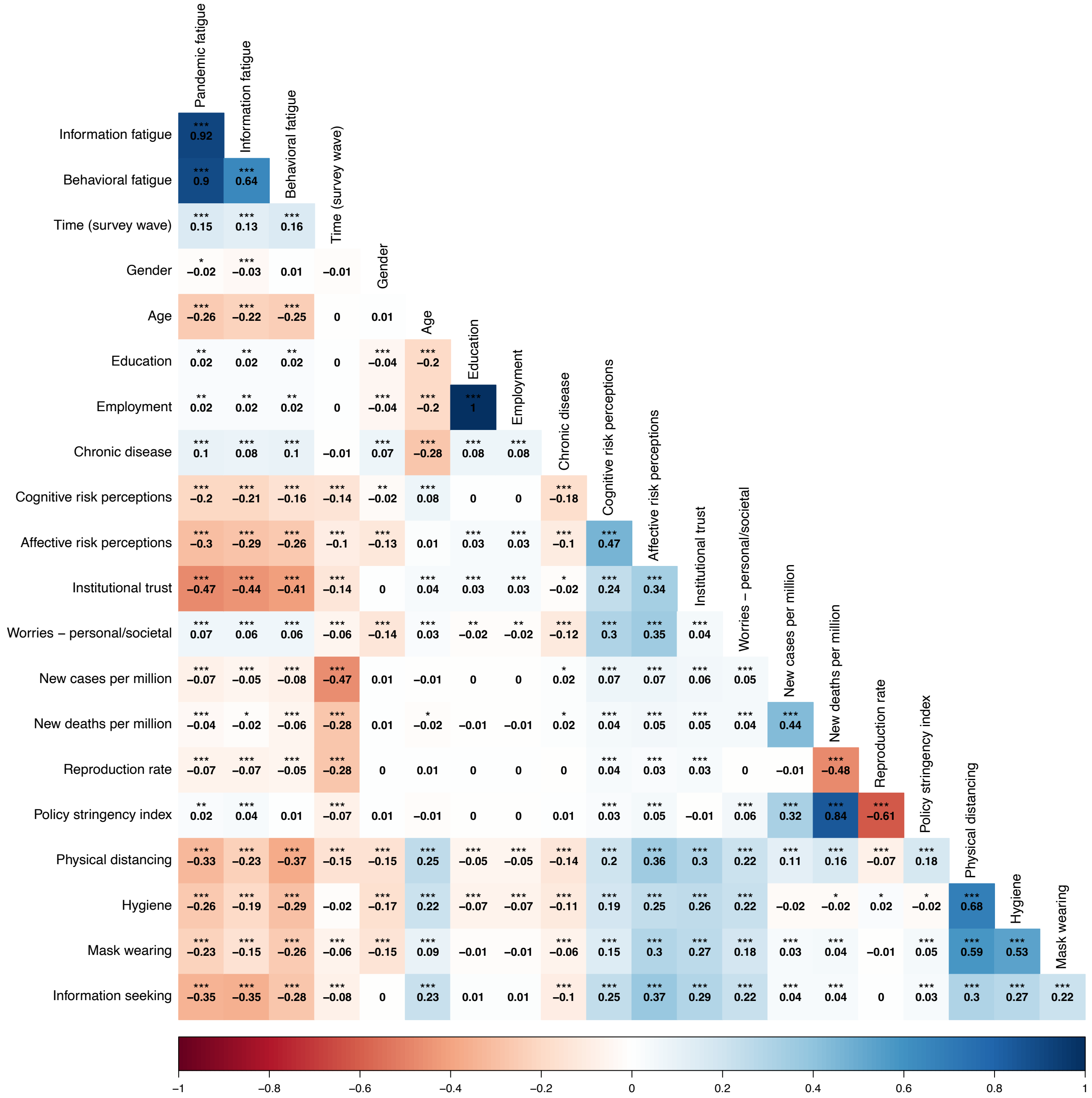

Note. For all correlations n ≤ 17,401 and n ≥ 6,281. Gender (male = 1, female = 0); Education (10 years or more = 1, less than 10 years = 0); Employment (unemployed = 1, employed = 0); Chronic disease (no = 1, yes = 0). The p-values have not been adjusted for multiple comparisons and are presented as follows: $^{***}p_{\text{two-tailed}} < .001$; $^{**}p_{\text{two-tailed}} < 0.01$; $^{*}p_{\text{two-tailed}} < 0.05$. The gender variable refers to participants self-identified gender as presented to them in the surveys.

Figure S4. Mixed-model regressions predicting physical distancing in Denmark.

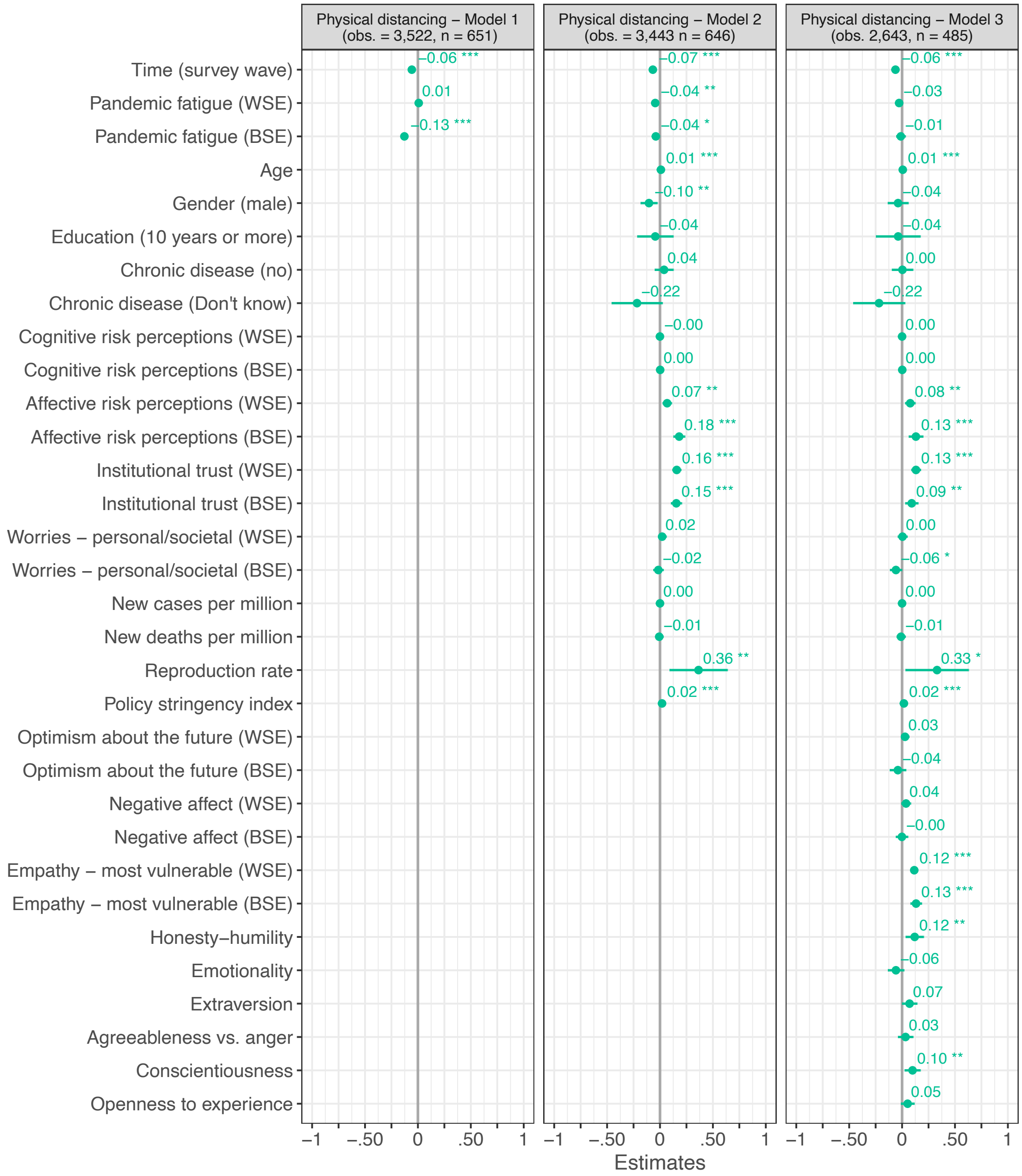

Note. Figure S4 shows estimated β coefficients with 95% confidence intervals based on mixed-model regressions with data from the Danish panel survey. Continuous time-invariant predictors as well as continuous time-varying contextual predictors (i.e., Time (survey wave), new cases per million, new deaths per million, reproduction rate, and policy stringency index) have been mean-centered. All other time-varying predictors have been centered using the person-mean centering approach to disaggregate the within- (WSE) and between-subjects effects (BSE) of these factors[21,22]. The p-values have not been adjusted for multiple comparisons and are presented as follows: $^{***}p_{\text{two-tailed}} < .001$; $^{**}p_{\text{two-tailed}} < 0.01$; $^{*}p_{\text{two-tailed}} < 0.05$. Exact p-values for all models are presented in the R-output which has been deposited on the Open Science Framework at: http://dx.doi.org/10.17605/OSF.IO/XD463. The gender variable refers to participants self-identified gender as presented to them in the surveys.

Figure S5. Mixed-model regressions predicting hygiene in Denmark.

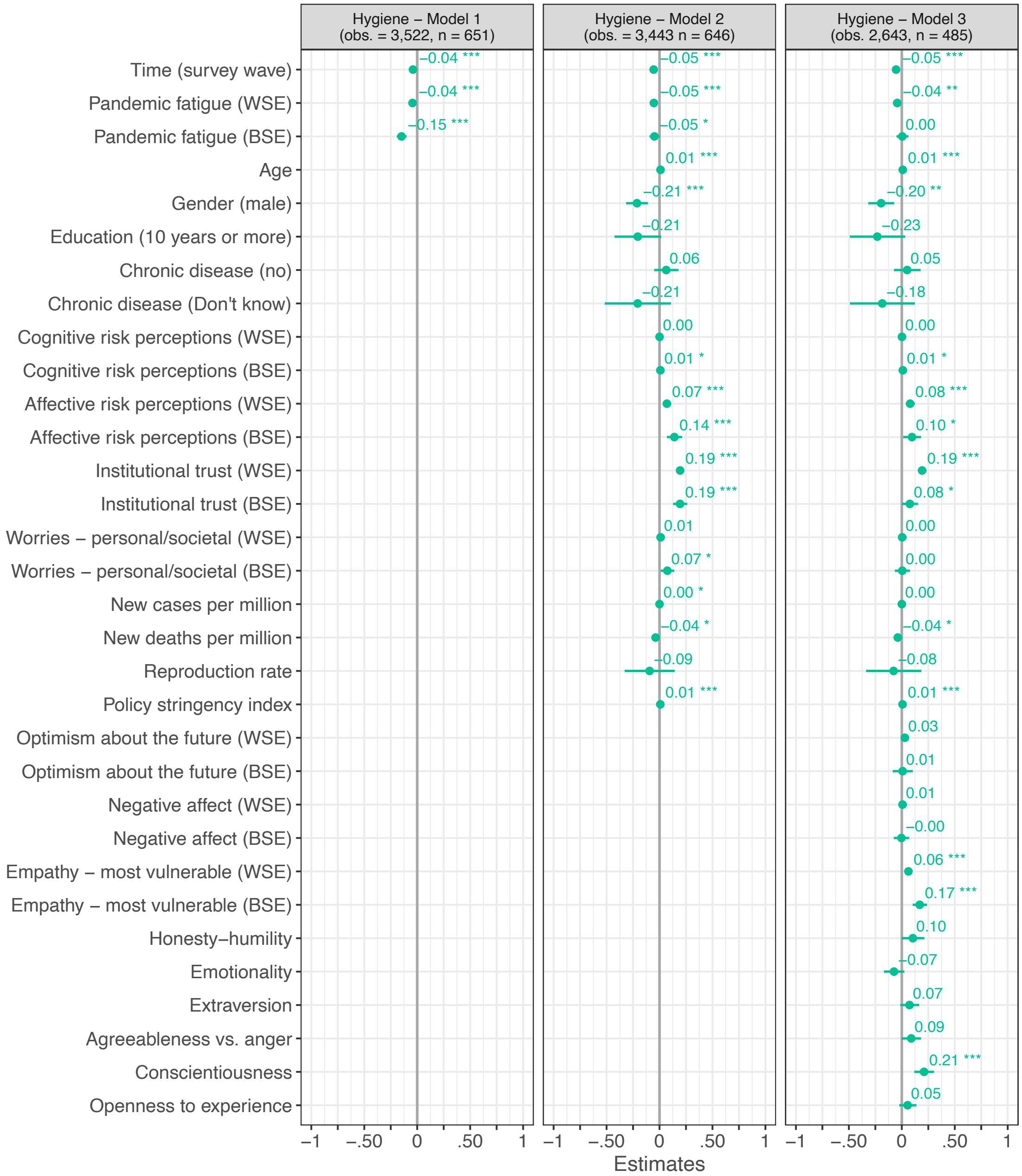

Note. Figure S5 shows estimated β coefficients with 95% confidence intervals based on mixed-model regressions with data from the Danish panel survey. Continuous time-invariant predictors as well as continuous time-varying contextual predictors (i.e., Time (survey wave), new cases per million, new deaths per million, reproduction rate, and policy stringency index) have been mean-centered. All other time-varying predictors have been centered using the person-mean centering approach to disaggregate the within- (WSE) and between-subjects effects (BSE) of these factors[21,22]. The p-values have not been adjusted for multiple comparisons and are presented as follows: $^{***}p_{\text{two-tailed}} < .001$; $^{**}p_{\text{two-tailed}} < 0.01$; $^{*}p_{\text{two-tailed}} < 0.05$. Exact p-values for all models are presented in the R-output which has been deposited on the Open Science Framework at: http://dx.doi.org/10.17605/OSF.IO/XD463. The gender variable refers to participants self-identified gender as presented to them in the surveys.

Figure S6. Mixed-model regressions predicting mask wearing in Denmark.

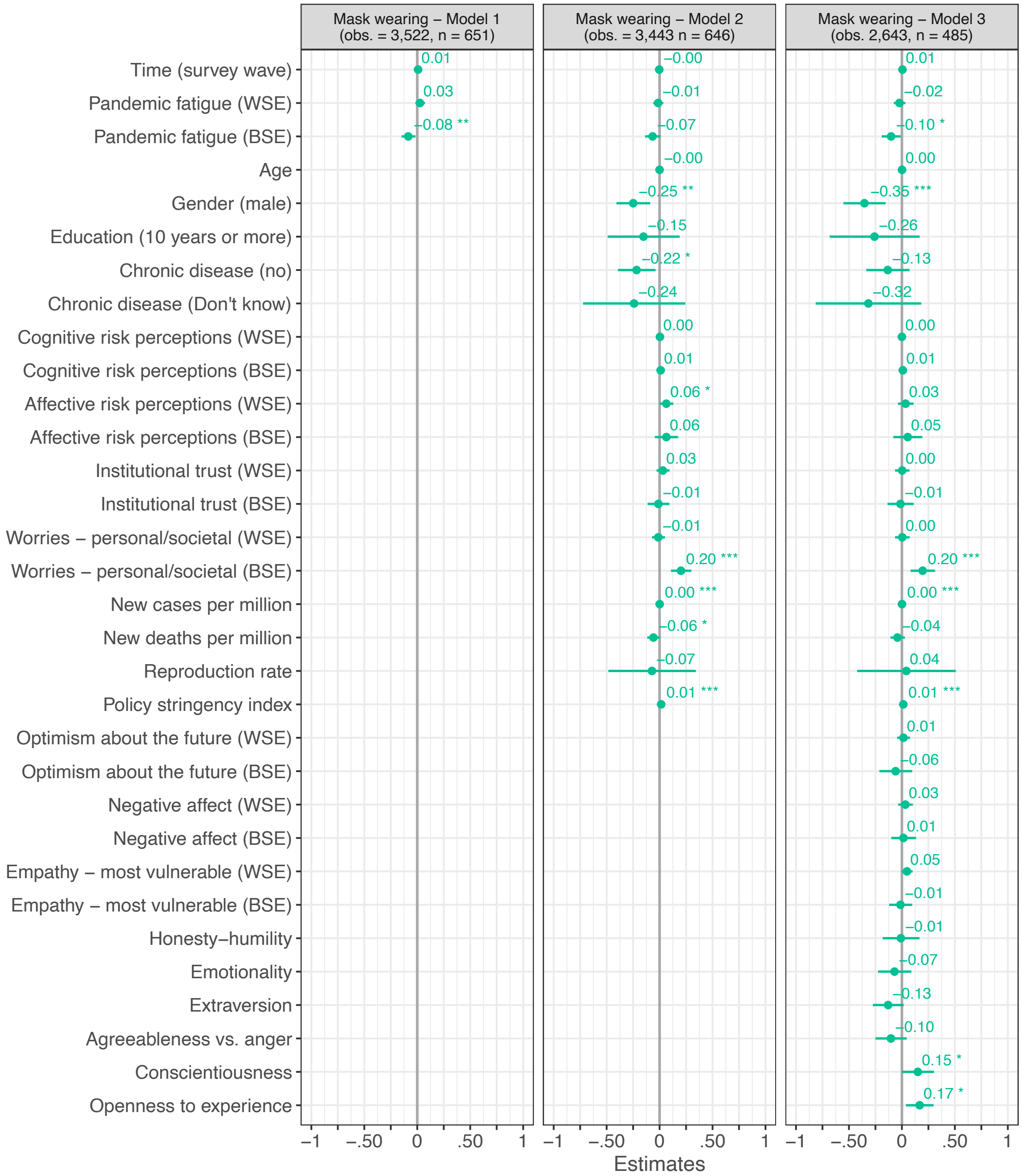

Note. Figure S6 shows estimated β coefficients with 95% confidence intervals based on mixed-model regressions with data from the Danish panel survey. Continuous time-invariant predictors as well as continuous time-varying contextual predictors (i.e., Time (survey wave), new cases per million, new deaths per million, reproduction rate, and policy stringency index) have been mean-centered. All other time-varying predictors have been centered using the person-mean centering approach to disaggregate the within- (WSE) and between-subjects effects (BSE) of these factors[21,22]. The p-values have not been adjusted for multiple comparisons and are presented as follows: $^{***}p_{\text{two-tailed}} < .001$; $^{**}p_{\text{two-tailed}} < 0.01$; $^{*}p_{\text{two-tailed}} < 0.05$. Exact p-values for all models are presented in the R-output which has been deposited on the Open Science Framework at: http://dx.doi.org/10.17605/OSF.IO/XD463. The gender variable refers to participants self-identified gender as presented to them in the surveys.

Figure S7. Mixed-model regressions predicting information seeking in Denmark.

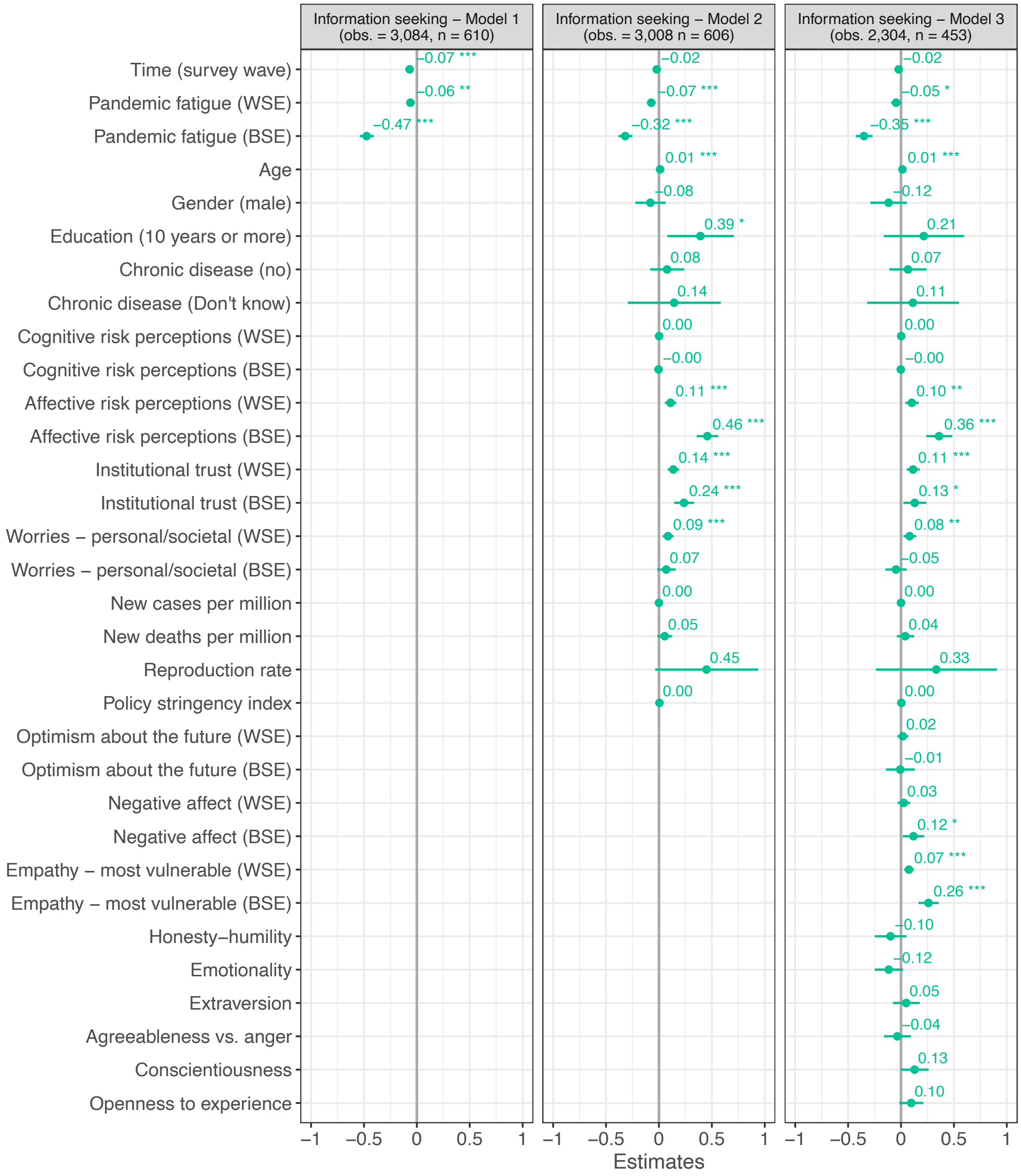

Note. Figure S7 shows estimated β coefficients with 95% confidence intervals based on mixed-model regressions with data from the Danish panel survey. Continuous time-invariant predictors as well as continuous time-varying contextual predictors (i.e., Time (survey wave), new cases per million, new deaths per million, reproduction rate, and policy stringency index) have been mean-centered. All other time-varying predictors have been centered using the person-mean centering approach to disaggregate the within- (WSE) and between-subjects effects (BSE) of these factors[21,22]. The p-values have not been adjusted for multiple comparisons and are presented as follows: $^{***}p_{\text{two-tailed}} < .001$; $^{**}p_{\text{two-tailed}} < 0.01$; $^{*}p_{\text{two-tailed}} < 0.05$. Exact p-values for all models are presented in the R-output which has been deposited on the Open Science Framework at: http://dx.doi.org/10.17605/OSF.IO/XD463. The gender variable refers to participants self-identified gender as presented to them in the surveys.

Figure S8. Self-reported public adherence to recommended health-protective behaviors over time in Denmark and Germany.

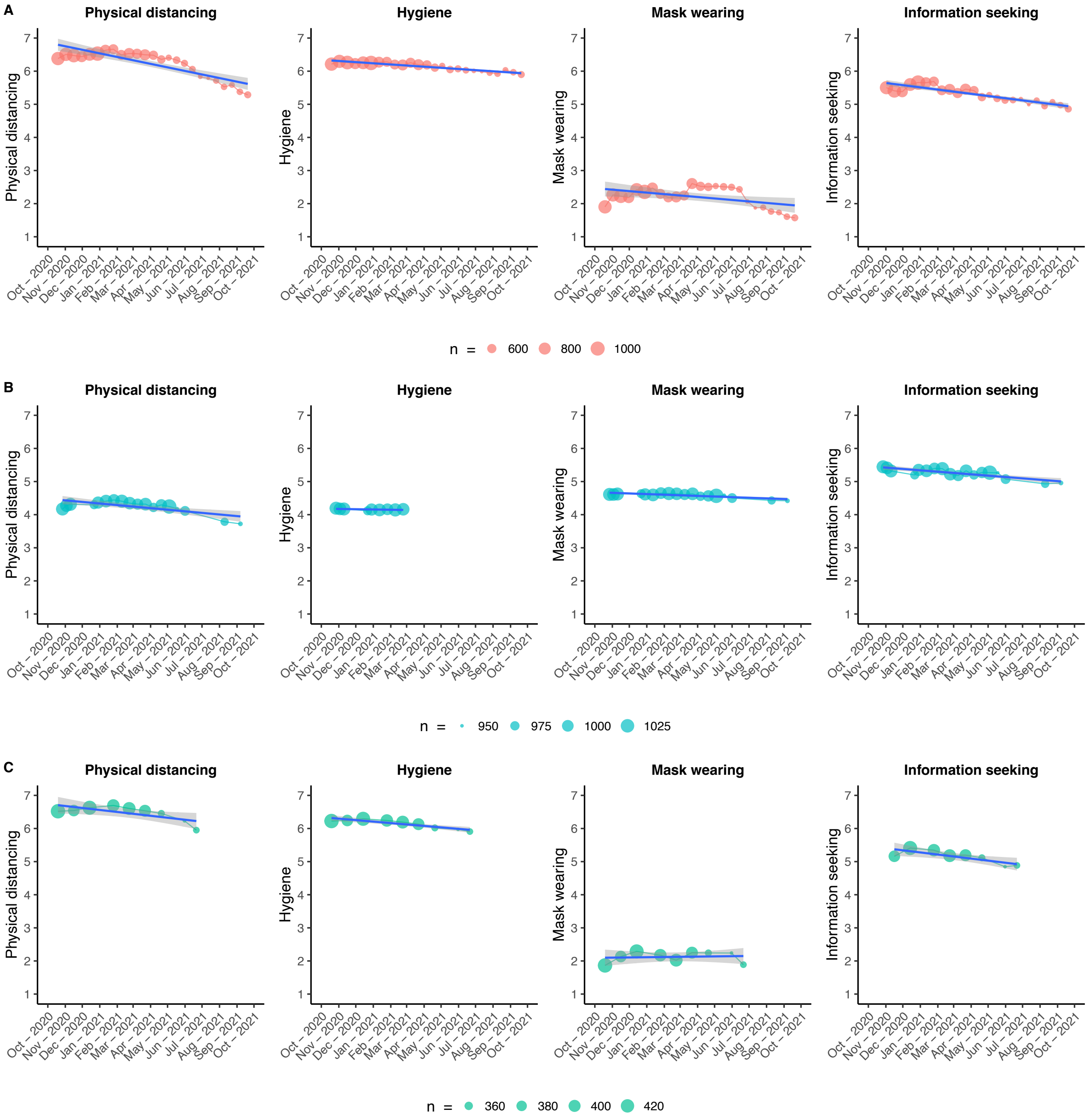

Note. Figure S8A, Figure S8B, and Figure S8C shows the mean level of self-reported public adherence to recommended health-protective behaviors for each wave of the Danish repeated cross-sectional survey, the German repeated cross-sectional survey, and the Danish panel survey, respectively, together with ordinary least square regression lines with 95% confidence intervals.

Figure S9. OLS regressions predicting information fatigue in Denmark and Germany.

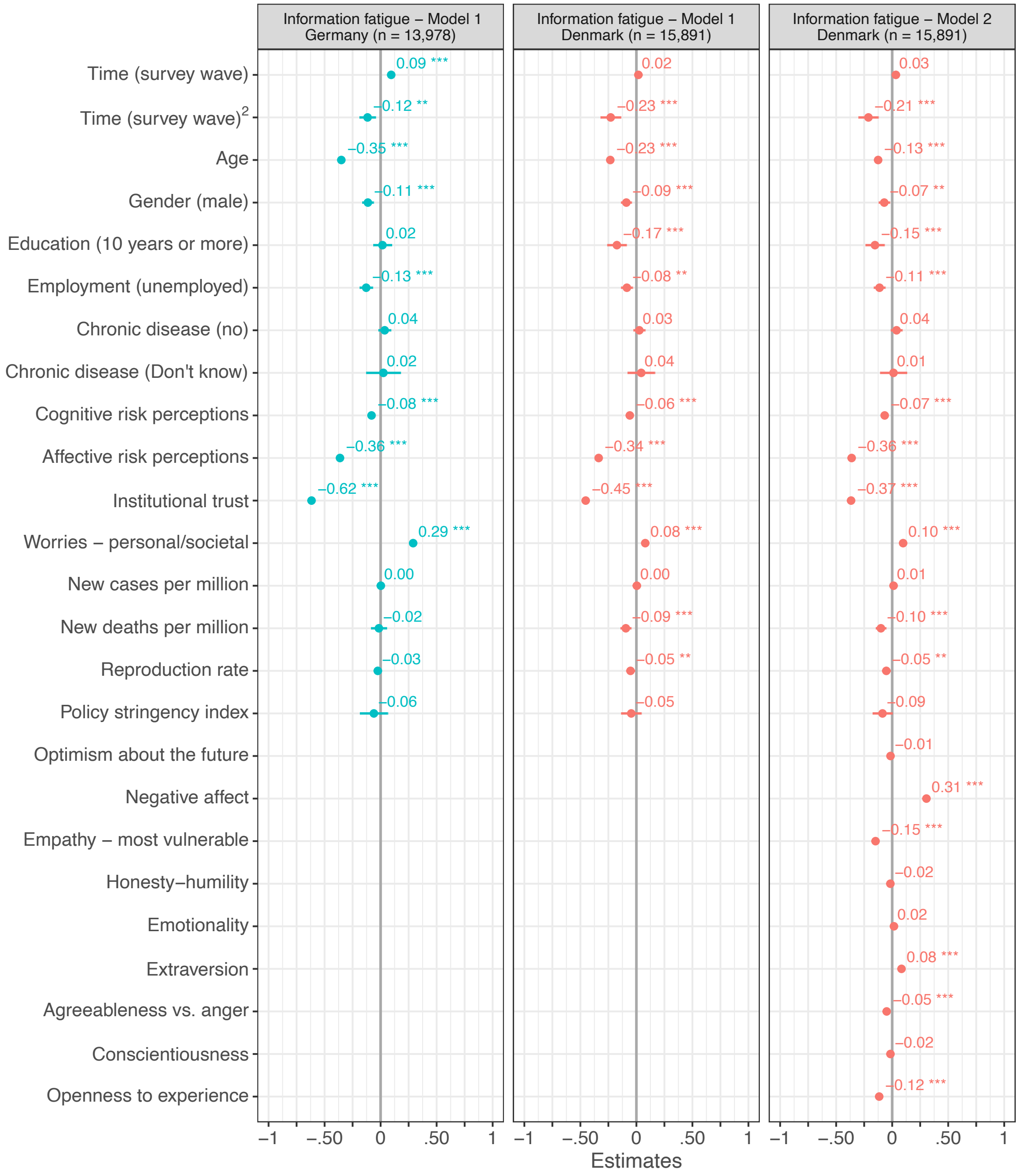

Note. Figure S9 shows standardized β coefficients with 95% confidence intervals based on ordinary least squares regressions with data from the Danish and German repeated cross-sectional surveys. All continuous predictors have been mean-centered and scaled by 1 standard deviation. The p-values have not been adjusted for multiple comparisons and are presented as follows: $^{***}p_{\text{two-tailed}} < .001$; $^{**}p_{\text{two-tailed}} < 0.01$; $^{*}p_{\text{two-tailed}} < 0.05$. Exact p-values for all models are presented in the R-output which has been deposited on the Open Science Framework at: http://dx.doi.org/10.17605/OSF.IO/XD463. The gender variable refers to participants self-identified gender as presented to them in the surveys. Participants who did not identify as either male or female are not included in the analyses due to an insufficient number of observations.

Figure S10. OLS regressions predicting behavioral fatigue in Denmark and Germany.

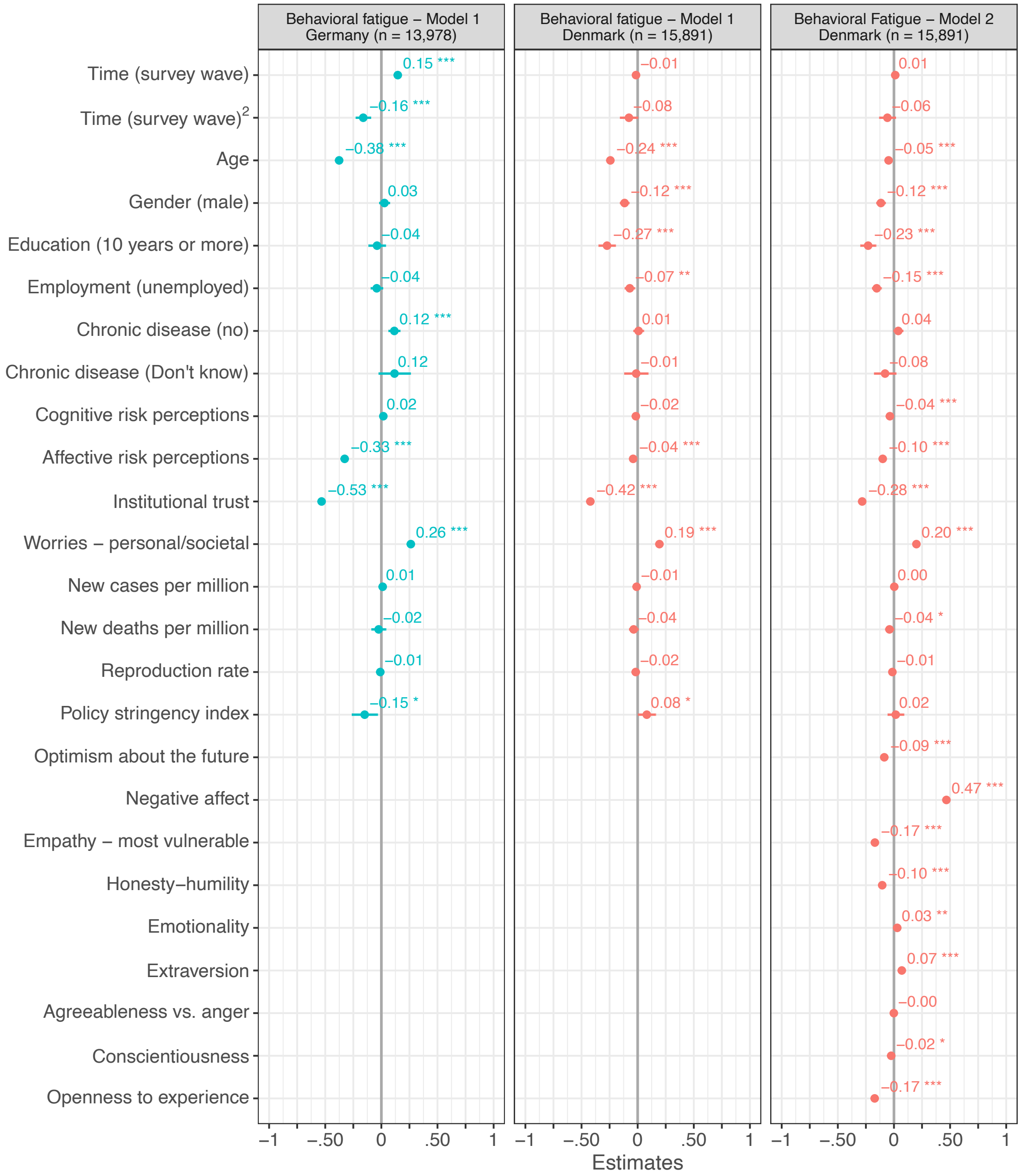

Note. Figure S10 shows standardized β coefficients with 95% confidence intervals based on ordinary least squares regressions with data from the Danish and German repeated cross-sectional surveys. All continuous predictors have been mean-centered and scaled by 1 standard deviation. The p-values have not been adjusted for multiple comparisons and are presented as follows: $^{***}p_{\text{two-tailed}} < .001$; $^{**}p_{\text{two-tailed}} < 0.01$; $^{*}p_{\text{two-tailed}} < 0.05$. Exact p-values for all models are presented in the R-output which has been deposited on the Open Science Framework at: http://dx.doi.org/10.17605/OSF.IO/XD463. The gender variable refers to participants self-identified gender as presented to them in the surveys. Participants who did not identify as either male or female are not included in the analyses due to an insufficient number of observations.

Figure S11. Mixed-model regressions predicting information fatigue in Denmark.

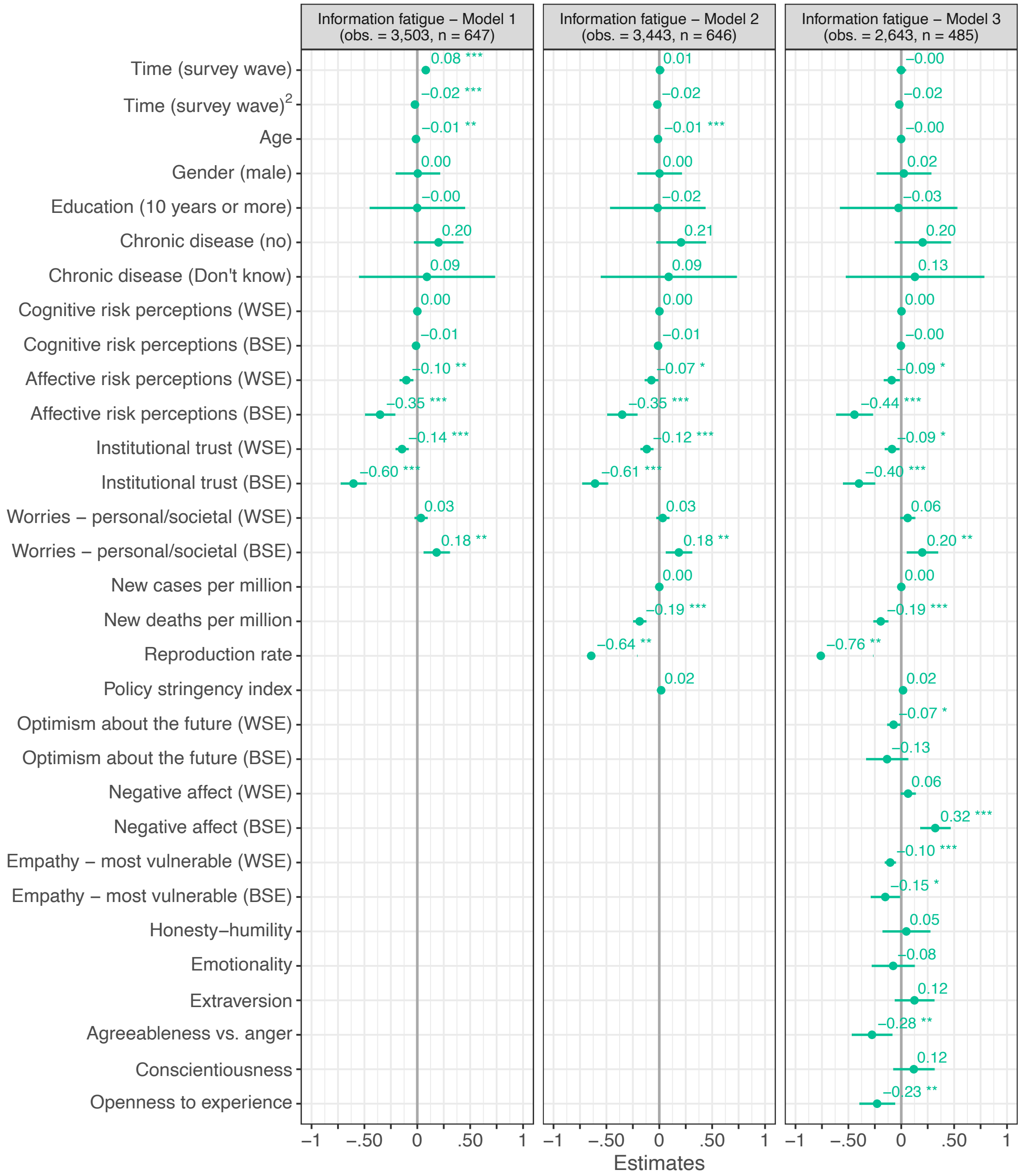

Note. Figure S11 shows estimated β coefficients with 95% confidence intervals based on mixed-model regressions with data from the Danish panel survey. Continuous time-invariant predictors as well as continuous time-varying contextual predictors (i.e., Time (survey wave), Time (survey wave)^2, new cases per million, new deaths per million, reproduction rate, and policy stringency index) have been mean-centered. All other time-varying predictors have been centered using the person-mean centering approach to disaggregate the within- (WSE) and between-subjects effects (BSE) of these factors[21,22]. The p-values have not been adjusted for multiple comparisons and are presented as follows: $^{***}p_{\text{two-tailed}} < .001$; $^{**}p_{\text{two-tailed}} < 0.01$; $^{*}p_{\text{two-tailed}} < 0.05$. Exact p-values for all models are presented in the R-output which has been deposited on the Open Science Framework at: http://dx.doi.org/10.17605/OSF.IO/XD463. The gender variable refers to participants self-identified gender as presented to them in the surveys.

Figure S12. Mixed-model regressions predicting behavioral fatigue in Denmark.

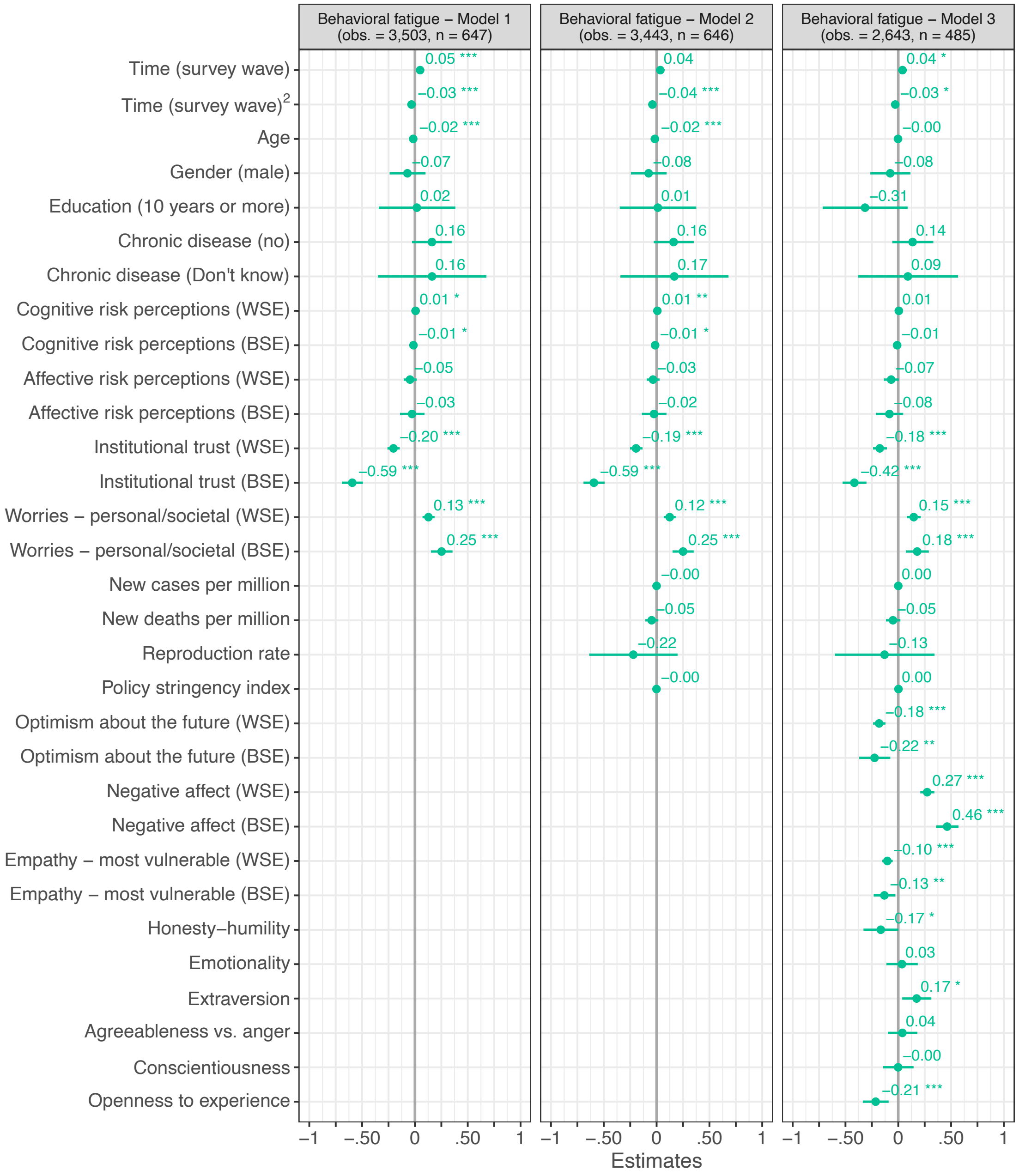

Note. Figure S12 shows estimated β coefficients with 95% confidence intervals based on mixed-model regressions with data from the Danish panel survey. Continuous time-invariant predictors as well as continuous time-varying contextual predictors (i.e., Time (survey wave), Time (survey wave)^2, new cases per million, new deaths per million, reproduction rate, and policy stringency index) have been mean-centered. All other time-varying predictors have been centered using the person-mean centering approach to disaggregate the within- (WSE) and between-subjects effects (BSE) of these factors[21,22]. The p-values have not been adjusted for multiple comparisons and are presented as follows: $^{***}p_{two\text{-}tailed} < .001$; $^{**}p_{two\text{-}tailed} < 0.01$; $^{*}p_{two\text{-}tailed} < 0.05$. Exact p-values for all models are presented in the R-output which has been deposited on the Open Science Framework at: http://dx.doi.org/10.17605/OSF.IO/XD463. The gender variable refers to participants self-identified gender as presented to them in the surveys.

Figure S13. OLS regressions predicting physical distancing in Denmark and Germany.

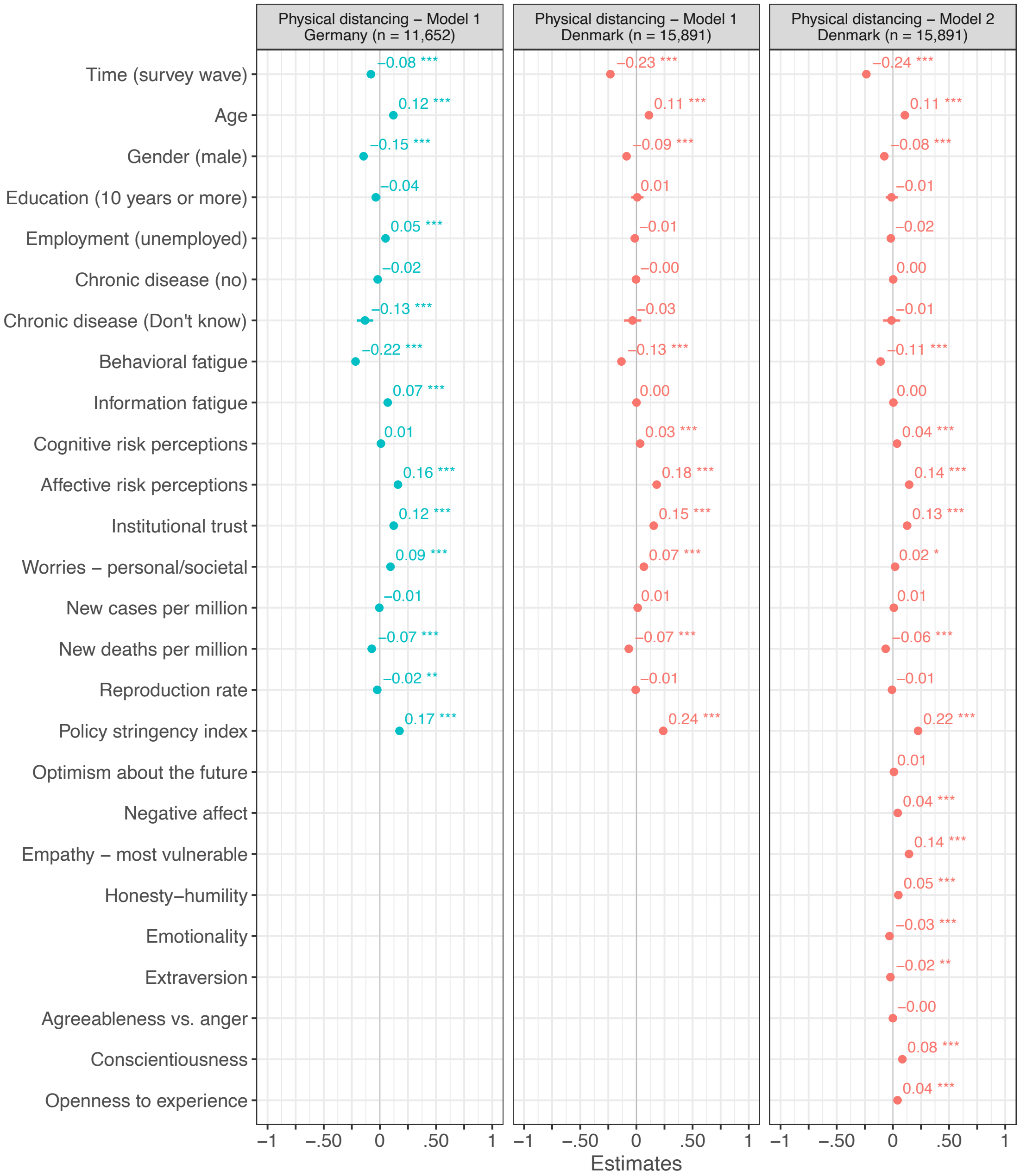

Note. Figure S13 shows standardized β coefficients with 95% confidence intervals based on ordinary least squares regressions with data from the Danish and German repeated cross-sectional surveys. All continuous predictors have been mean-centered and scaled by 1 standard deviation. The p-values have not been adjusted for multiple comparisons and are presented as follows: $^{***}p_{\text{two-tailed}} < .001$; $^{**}p_{\text{two-tailed}} < 0.01$; $^{*}p_{\text{two-tailed}} < 0.05$. Exact p-values for all models are presented in the R-output which has been deposited on the Open Science Framework at: http://dx.doi.org/10.17605/OSF.IO/XD463. The gender variable refers to participants self-identified gender as presented to them in the surveys. Participants who did not identify as either male or female are not included in the analyses due to an insufficient number of observations.

Figure S14. OLS regressions predicting hygiene in Denmark and Germany.

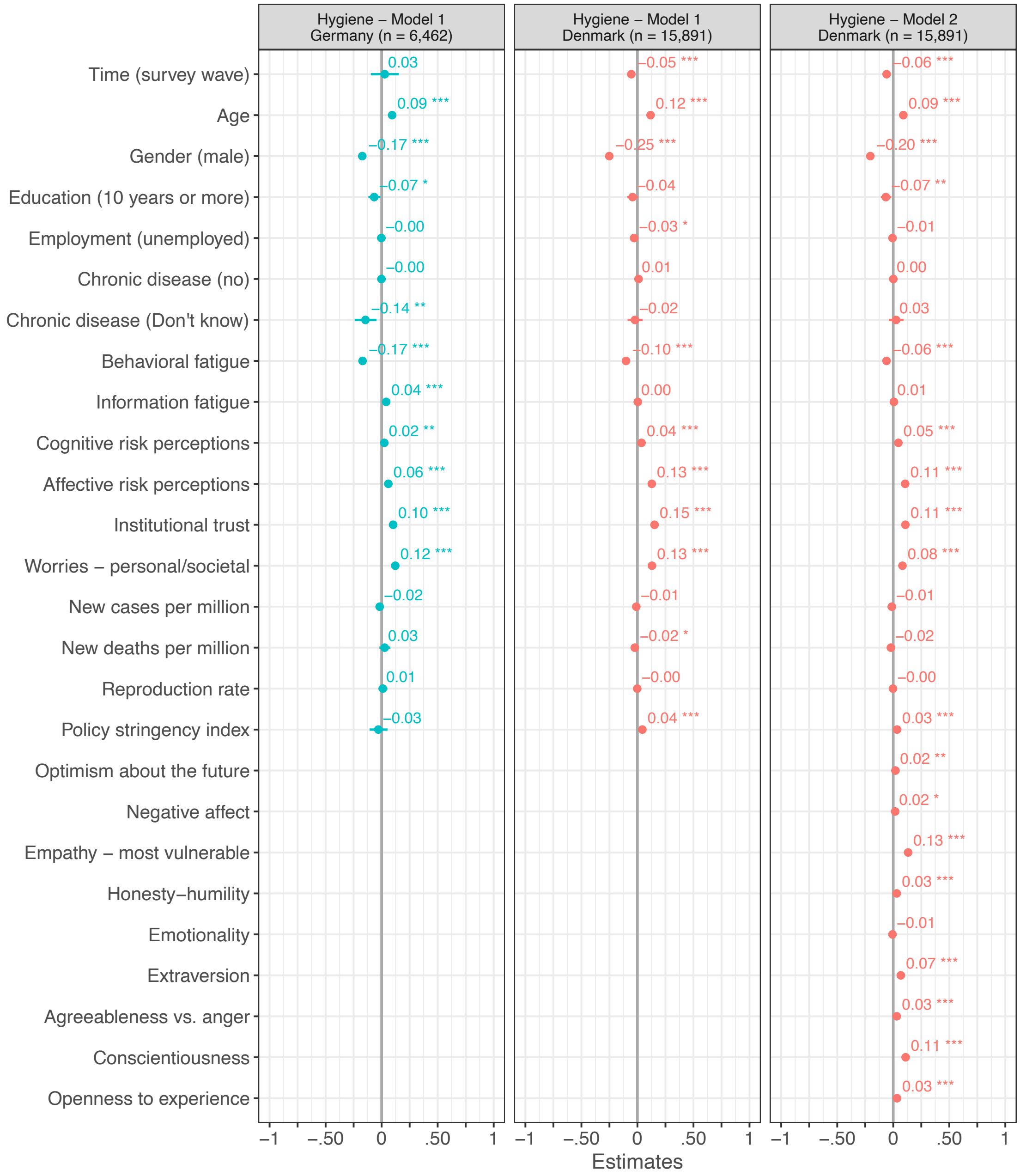

Note. Figure S14 shows standardized β coefficients with 95% confidence intervals based on ordinary least squares regressions with data from the Danish and German repeated cross-sectional surveys. All continuous predictors have been mean-centered and scaled by 1 standard deviation. The p-values have not been adjusted for multiple comparisons and are presented as follows: $^{***}p_{\text{two-tailed}} < .001$; $^{**}p_{\text{two-tailed}} < 0.01$; $^{*}p_{\text{two-tailed}} < 0.05$. Exact p-values for all models are presented in the R-output which has been deposited on the Open Science Framework at: http://dx.doi.org/10.17605/OSF.IO/XD463. The gender variable refers to participants self-identified gender as presented to them in the surveys. Participants who did not identify as either male or female are not included in the analyses due to an insufficient number of observations.

Figure S15. OLS regressions predicting mask wearing in Denmark and Germany.

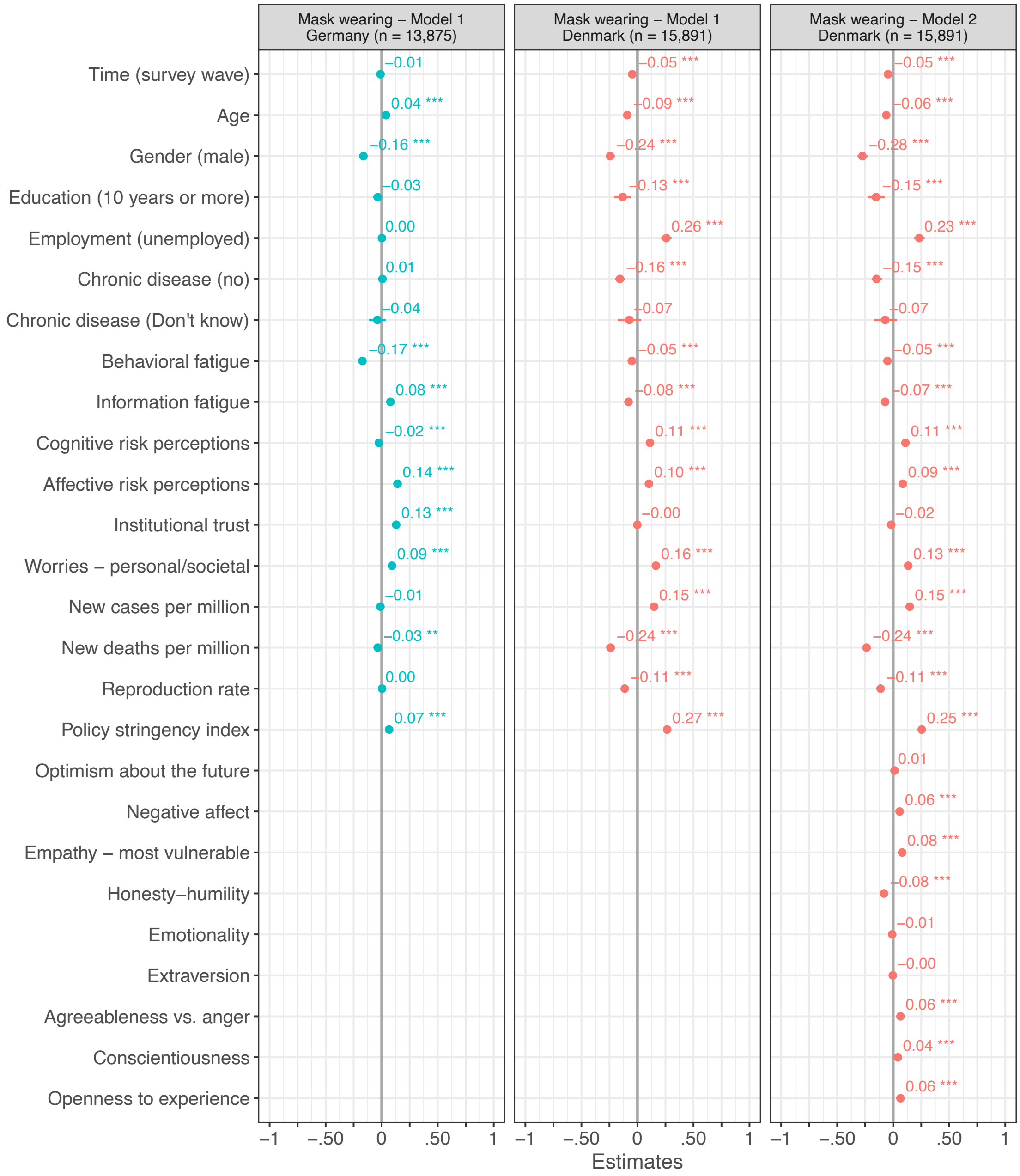

Note. Figure S15 shows standardized β coefficients with 95% confidence intervals based on ordinary least squares regressions with data from the Danish and German repeated cross-sectional surveys. All continuous predictors have been mean-centered and scaled by 1 standard deviation. The p-values have not been adjusted for multiple comparisons and are presented as follows: $^{***}p_{\text{two-tailed}} < .001$; $^{**}p_{\text{two-tailed}} < 0.01$; $^{*}p_{\text{two-tailed}} < 0.05$. Exact p-values for all models are presented in the R-output which has been deposited on the Open Science Framework at: http://dx.doi.org/10.17605/OSF.IO/XD463. The gender variable refers to participants self-identified gender as presented to them in the surveys. Participants who did not identify as either male or female are not included in the analyses due to an insufficient number of observations.

Figure S16. OLS regressions predicting information seeking in Denmark and Germany.

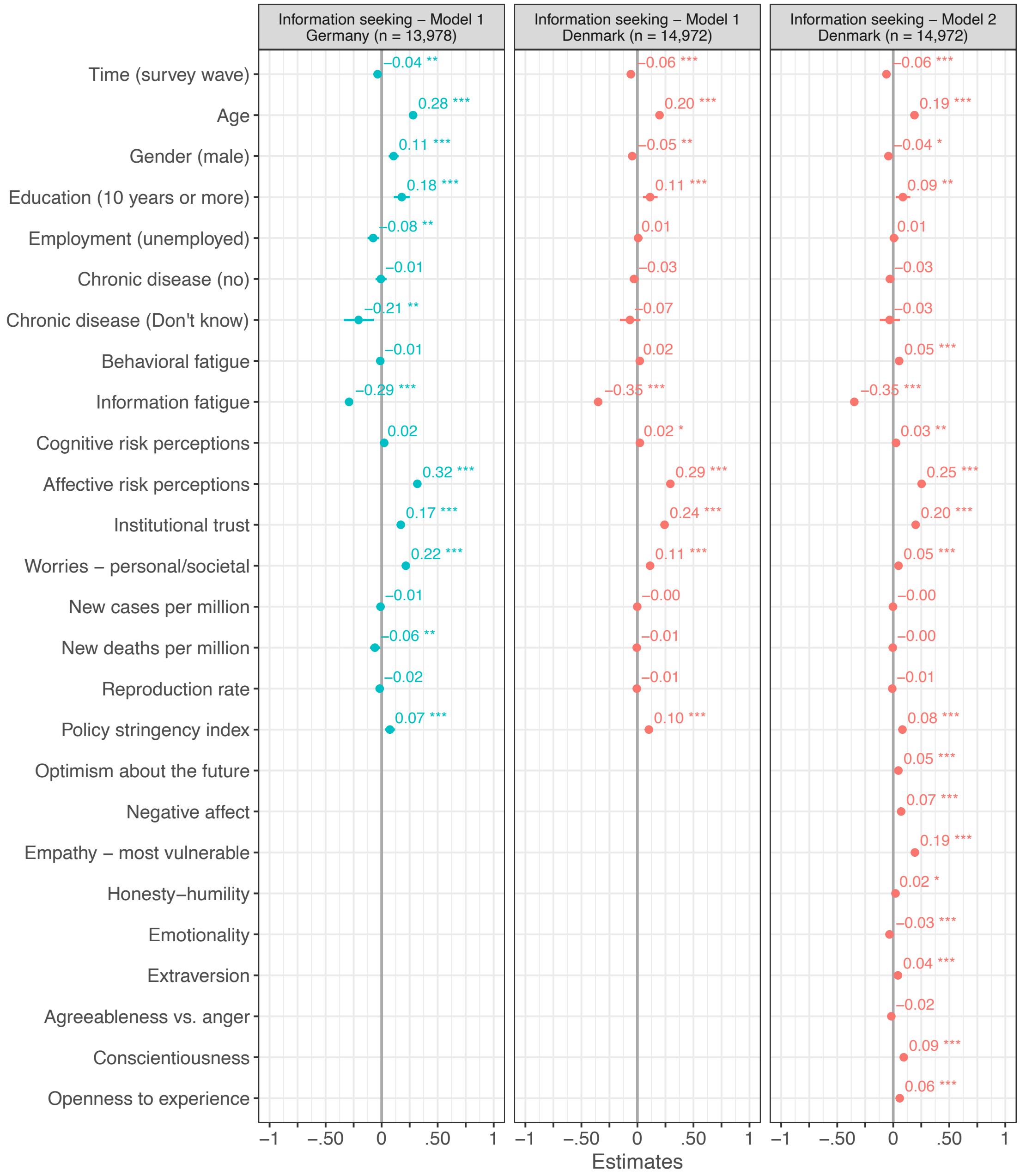

Note. Figure S16 shows standardized β coefficients with 95% confidence intervals based on ordinary least squares regressions with data from the Danish and German repeated cross-sectional surveys. All continuous predictors have been mean-centered and scaled by 1 standard deviation. The p-values have not been adjusted for multiple comparisons and are presented as follows: $^{***}p_{\text{two-tailed}} < .001$; $^{**}p_{\text{two-tailed}} < 0.01$; $^{*}p_{\text{two-tailed}} < 0.05$. Exact p-values for all models are presented in the R-output which has been deposited on the Open Science Framework at: http://dx.doi.org/10.17605/OSF.IO/XD463. The gender variable refers to participants self-identified gender as presented to them in the surveys. Participants who did not identify as either male or female are not included in the analyses due to an insufficient number of observations.

Figure S17. Mixed-model regressions predicting physical distancing in Denmark.

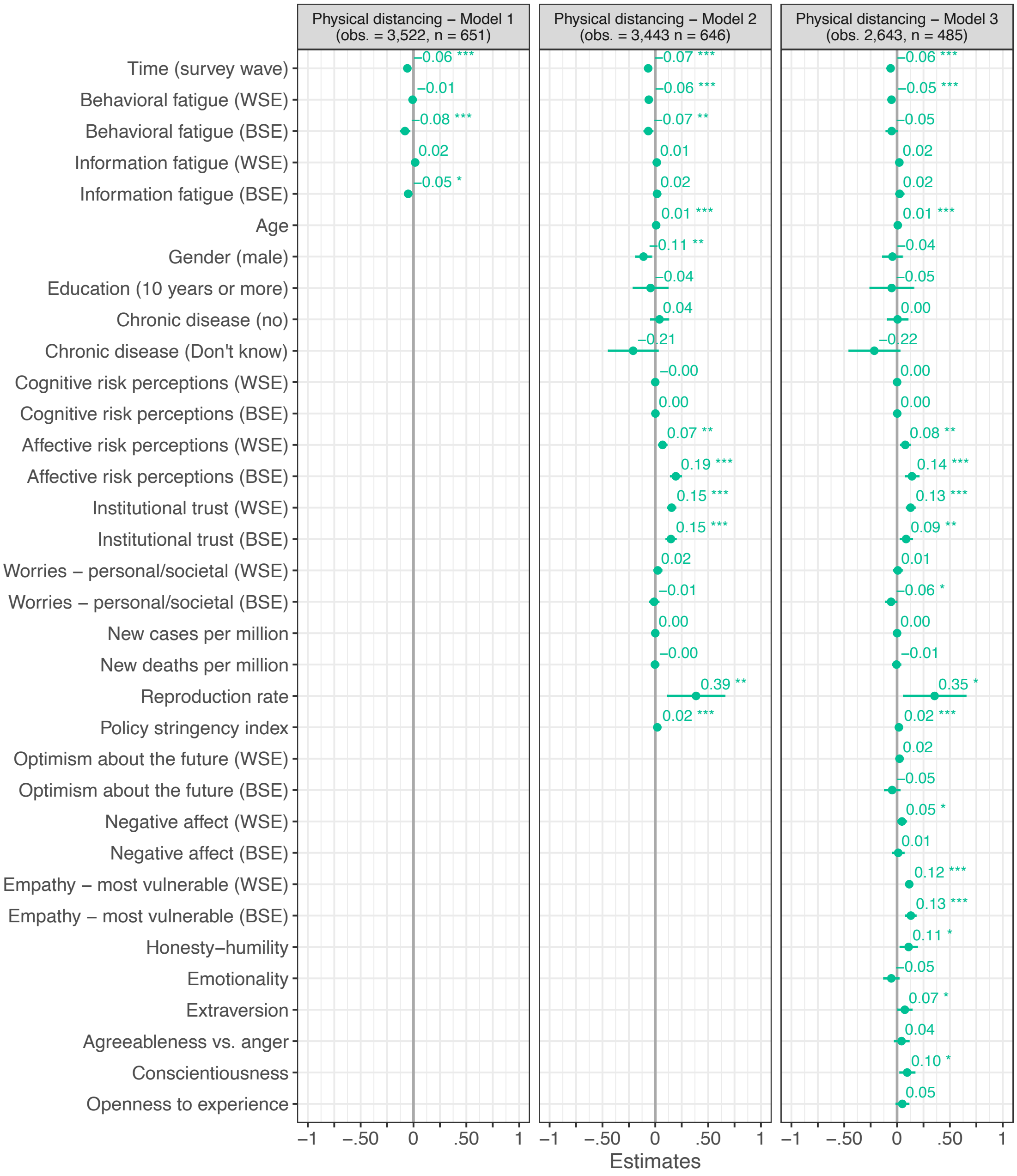

Note. Figure S17 shows estimated β coefficients with 95% confidence intervals based on mixed-model regressions with data from the Danish panel survey. Continuous time-invariant predictors as well as continuous time-varying contextual predictors (i.e., Time (survey wave), new cases per million, new deaths per million, reproduction rate, and policy stringency index) have been mean-centered. All other time-varying predictors have been centered using the person-mean centering approach to disaggregate the within- (WSE) and between-subjects effects (BSE) of these factors[21,22]. The p-values have not been adjusted for multiple comparisons and are presented as follows: $^{***}p_{\text{two-tailed}} < .001$; $^{**}p_{\text{two-tailed}} < 0.01$; $^{*}p_{\text{two-tailed}} < 0.05$. Exact p-values for all models are presented in the R-output which has been deposited on the Open Science Framework at: http://dx.doi.org/10.17605/OSF.IO/XD463. The gender variable refers to participants self-identified gender as presented to them in the surveys.

Figure S18. Mixed-model regressions predicting hygiene in Denmark.

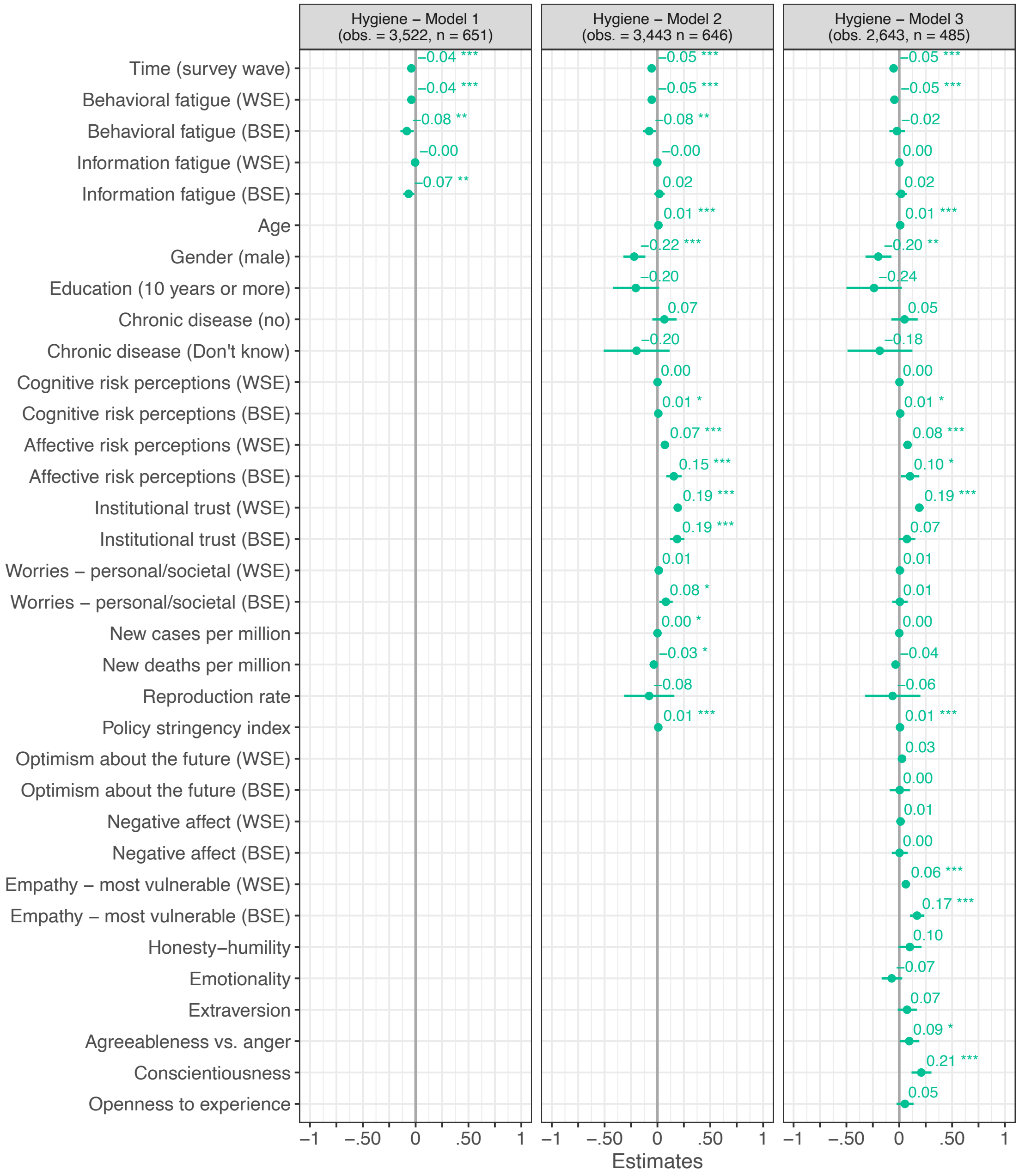

Note. Figure S18 shows estimated β coefficients with 95% confidence intervals based on mixed-model regressions with data from the Danish panel survey. Continuous time-invariant predictors as well as continuous time-varying contextual predictors (i.e., Time (survey wave), new cases per million, new deaths per million, reproduction rate, and policy stringency index) have been mean-centered. All other time-varying predictors have been centered using the person-mean centering approach to disaggregate the within- (WSE) and between-subjects effects (BSE) of these factors[21,22]. The p-values have not been adjusted for multiple comparisons and are presented as follows: $^{***}p_{\text{two-tailed}} < .001$; $^{**}p_{\text{two-tailed}} < 0.01$; $^{*}p_{\text{two-tailed}} < 0.05$. Exact p-values for all models are presented in the R-output which has been deposited on the Open Science Framework at: http://dx.doi.org/10.17605/OSF.IO/XD463. The gender variable refers to participants self-identified gender as presented to them in the surveys.

Figure S19. Mixed-model regressions predicting mask wearing in Denmark.

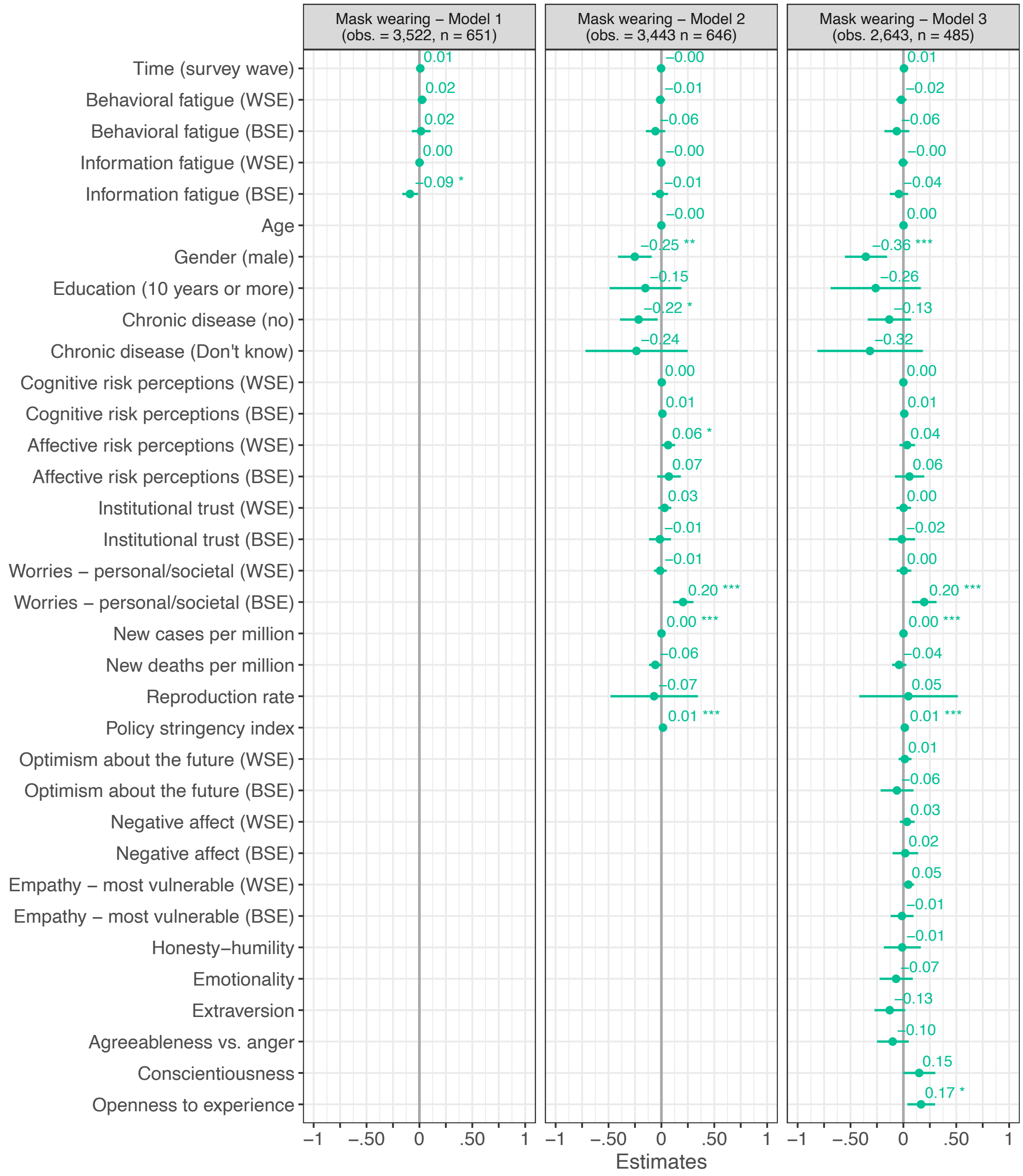

Note. Figure S19 shows estimated β coefficients with 95% confidence intervals based on mixed-model regressions with data from the Danish panel survey. Continuous time-invariant predictors as well as continuous time-varying contextual predictors (i.e., Time (survey wave), new cases per million, new deaths per million, reproduction rate, and policy stringency index) have been mean-centered. All other time-varying predictors have been centered using the person-mean centering approach to disaggregate the within- (WSE) and between-subjects effects (BSE) of these factors[21,22]. The p-values have not been adjusted for multiple comparisons and are presented as follows: $^{***}p_{\text{two-tailed}} < .001$; $^{**}p_{\text{two-tailed}} < 0.01$; $^{*}p_{\text{two-tailed}} < 0.05$. Exact p-values for all models are presented in the R-output which has been deposited on the Open Science Framework at: http://dx.doi.org/10.17605/OSF.IO/XD463. The gender variable refers to participants self-identified gender as presented to them in the surveys.

Figure S20. Mixed-model regressions predicting information seeking in Denmark.

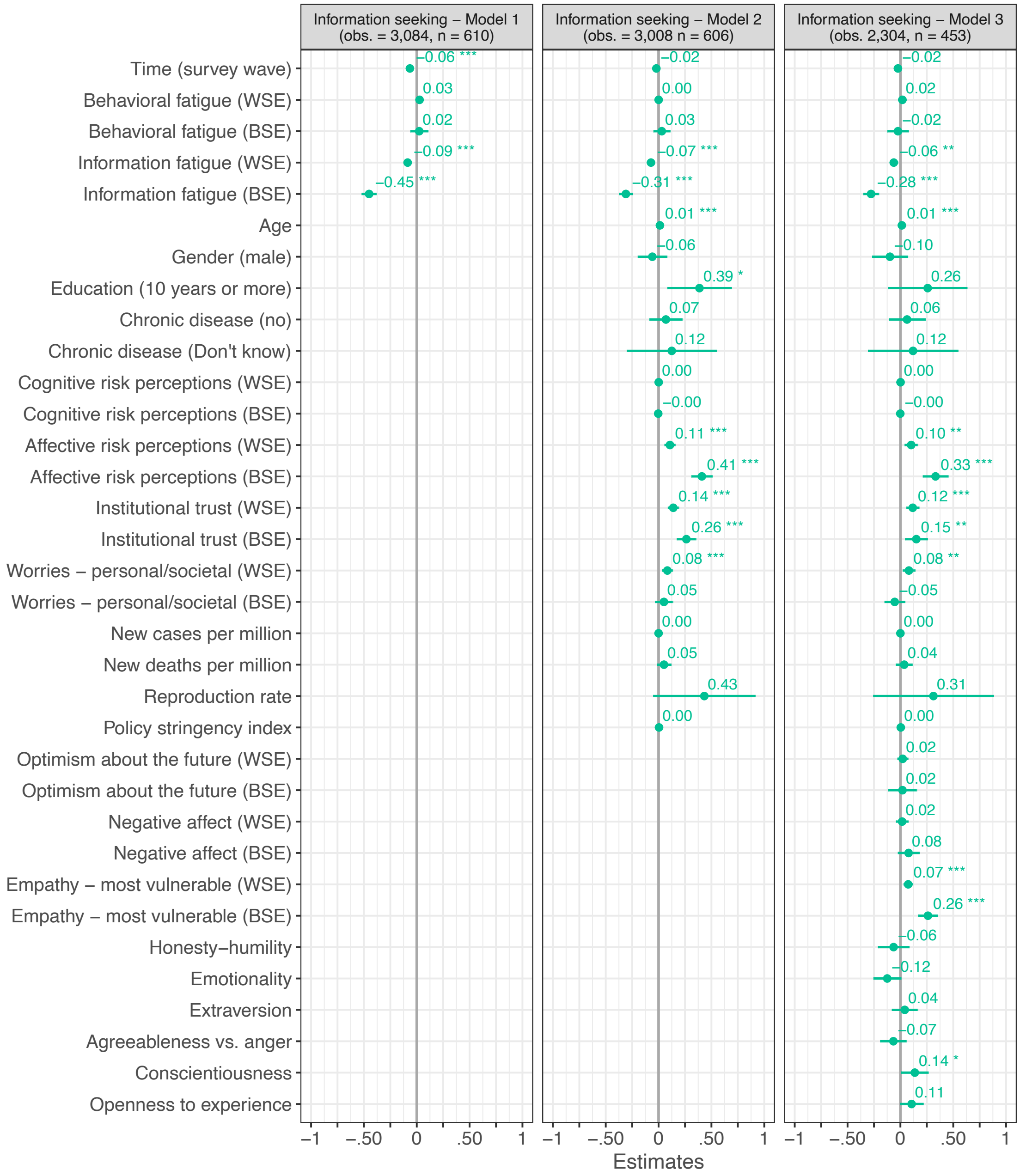

Note. Figure S20 shows estimated β coefficients with 95% confidence intervals based on mixed-model regressions with data from the Danish panel survey. Continuous time-invariant predictors as well as continuous time-varying contextual predictors (i.e., Time (survey wave), new cases per million, new deaths per million, reproduction rate, and policy stringency index) have been mean-centered. All other time-varying predictors have been centered using the person-mean centering approach to disaggregate the within- (WSE) and between-subjects effects (BSE) of these factors[21,22]. The p-values have not been adjusted for multiple comparisons and are presented as follows: $^{***}p_{\text{two-tailed}} < .001$; $^{**}p_{\text{two-tailed}} < 0.01$; $^{*}p_{\text{two-tailed}} < 0.05$. Exact p-values for all models are presented in the R-output which has been deposited on the Open Science Framework at: http://dx.doi.org/10.17605/OSF.IO/XD463. The gender variable refers to participants self-identified gender as presented to them in the surveys.

Figure S21. Scree plot and Glorfeld's modified parallel analysis.

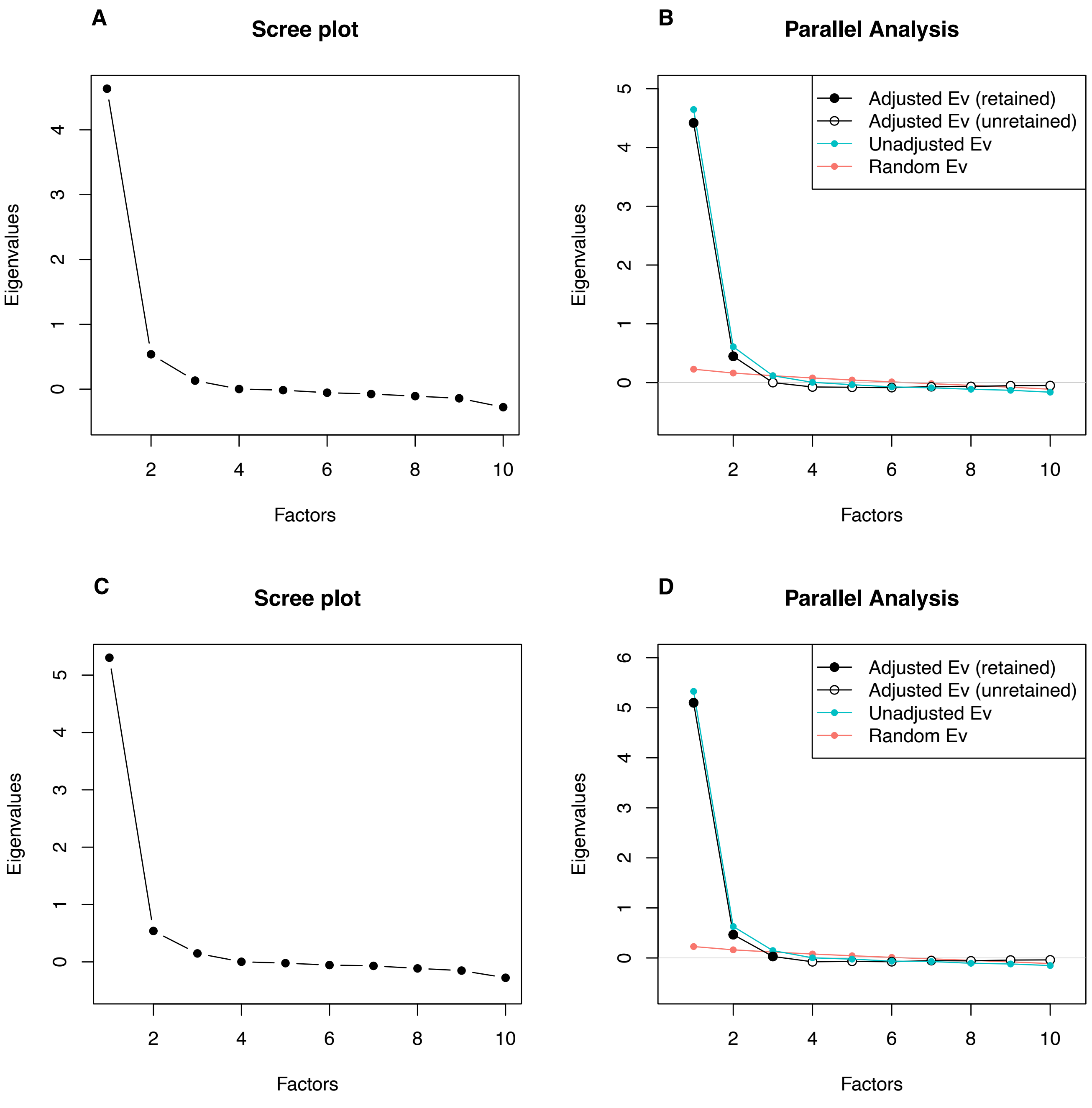

Note. Figure S21A shows the results from a scree test[9] based on Pearson's product-moment correlations. Figure S21B shows the results from Glorfeld's modified parallel analysis[10,11] based on Pearson's product-moment correlations. Figure S21C shows the results from a scree test[9] based on polychoric correlations. Figure S21D shows the results from Glorfeld's modified parallel analysis[10,11] based on polychoric correlations. Abbreviations: Eigenvalue (Ev).

Figure S22. OLS regressions predicting adherence to health-protective behaviors in the U.S.

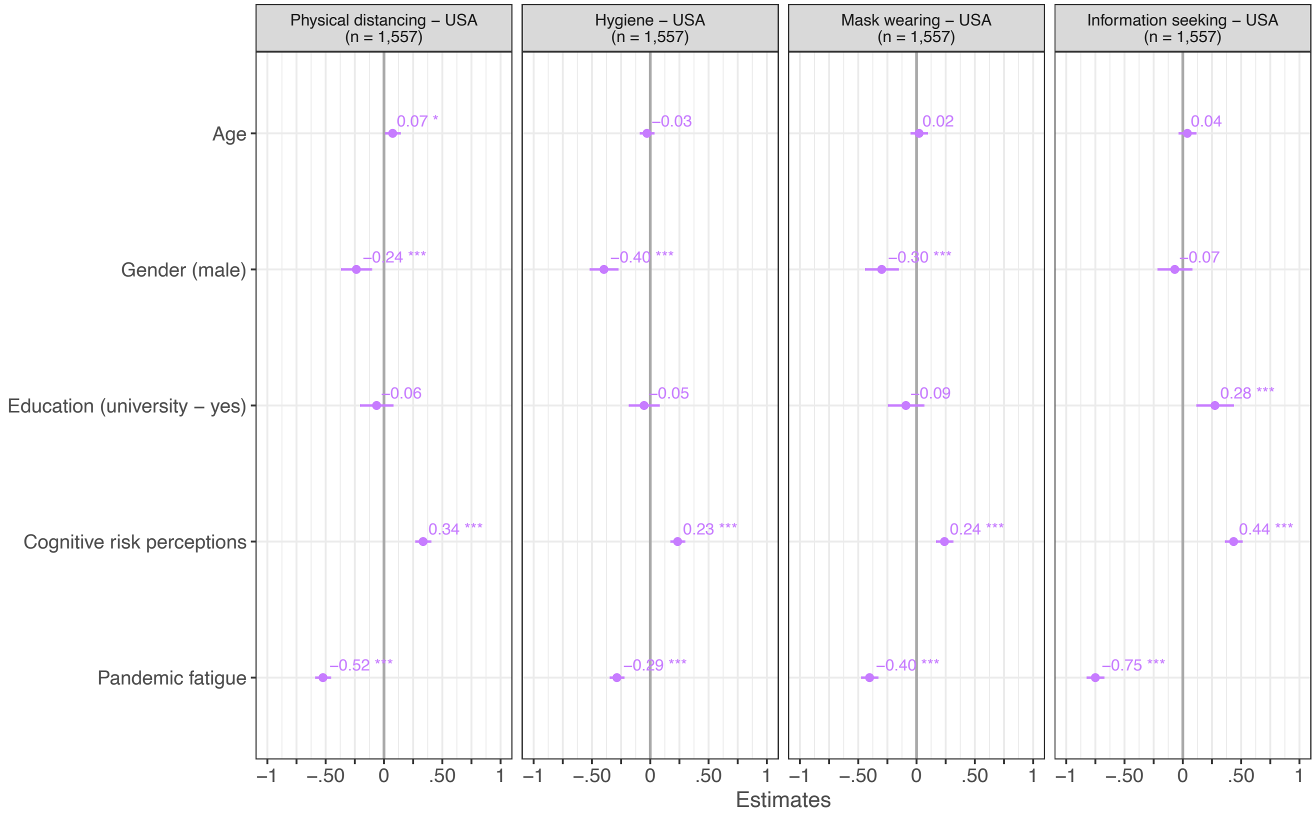

Note. Figure S22 shows standardized β coefficients with 95% confidence intervals based on ordinary least squares regressions with data from the experiment. All continuous predictors have been mean-centered and scaled by 1 standard deviation. The p-values have not been adjusted for multiple comparisons and are presented as follows: $^{***}p_{\text{two-tailed}} < .001$; $^{**}p_{\text{two-tailed}} < 0.01$; $^{*}p_{\text{two-tailed}} < 0.05$. Exact p-values for all models are presented in the R-output which has been deposited on the Open Science Framework at: http://dx.doi.org/10.17605/OSF.IO/XD463. The gender variable refers to participants self-identified gender as presented to them in the experiment. Participants who did not identify as either male or female are not included in the analyses due to an insufficient number of observations.

Figure S23. OLS regressions predicting adherence to health-protective behaviors in the U.S.

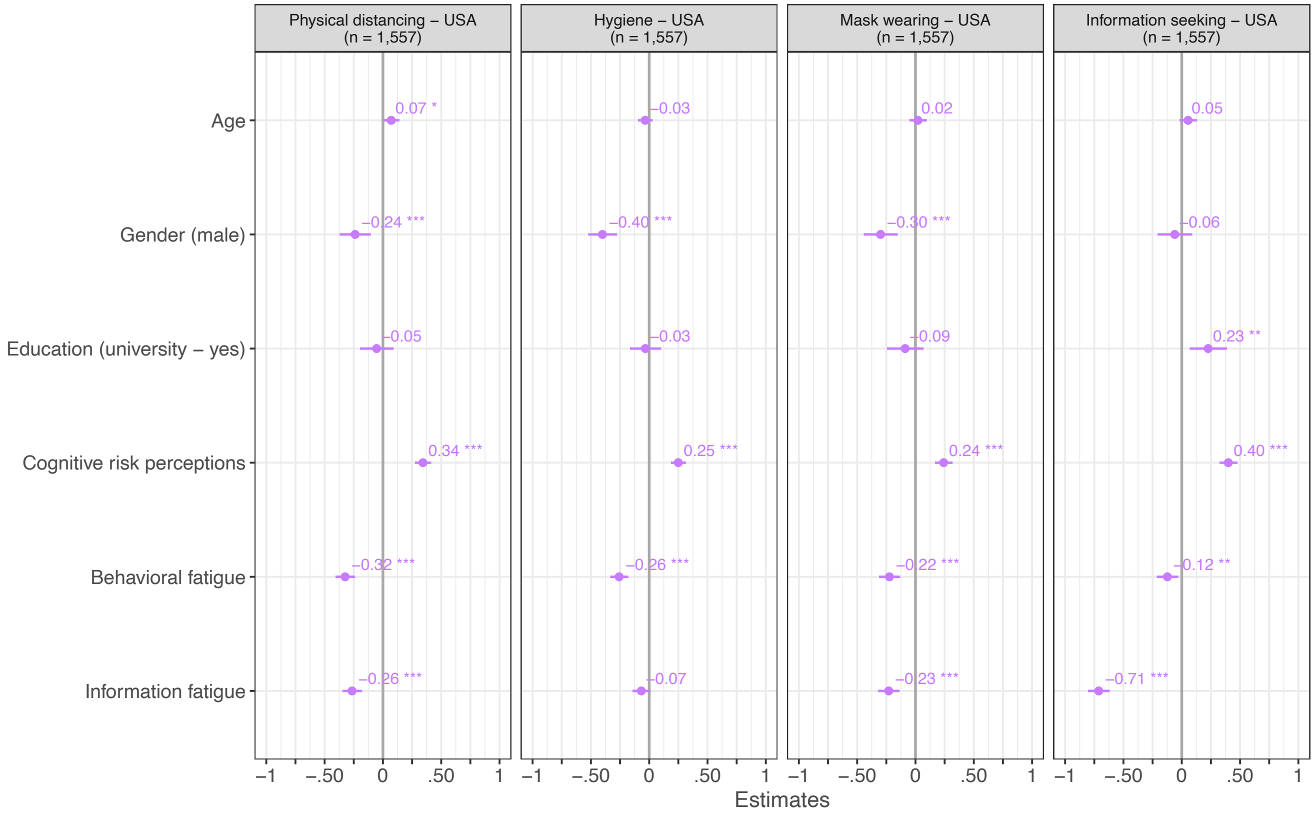

Note. Figure S23 shows standardized β coefficients with 95% confidence intervals based on ordinary least squares regressions with data from the experiment. All continuous predictors have been mean-centered and scaled by 1 standard deviation. The p-values have not been adjusted for multiple comparisons and are presented as follows: $^{***}p_{\text{two-tailed}} < .001$; $^{**}p_{\text{two-tailed}} < 0.01$; $^{*}p_{\text{two-tailed}} < 0.05$. Exact p-values for all models are presented in the R-output which has been deposited on the Open Science Framework at: http://dx.doi.org/10.17605/OSF.IO/XD463. The gender variable refers to participants self-identified gender as presented to them in the experiment. Participants who did not identify as either male or female are not included in the analyses due to an insufficient number of observations.

Figure S24. Two-factor and second-order models of pandemic fatigue fitted with robust diagonally weighted least squares estimation in Denmark and Germany.

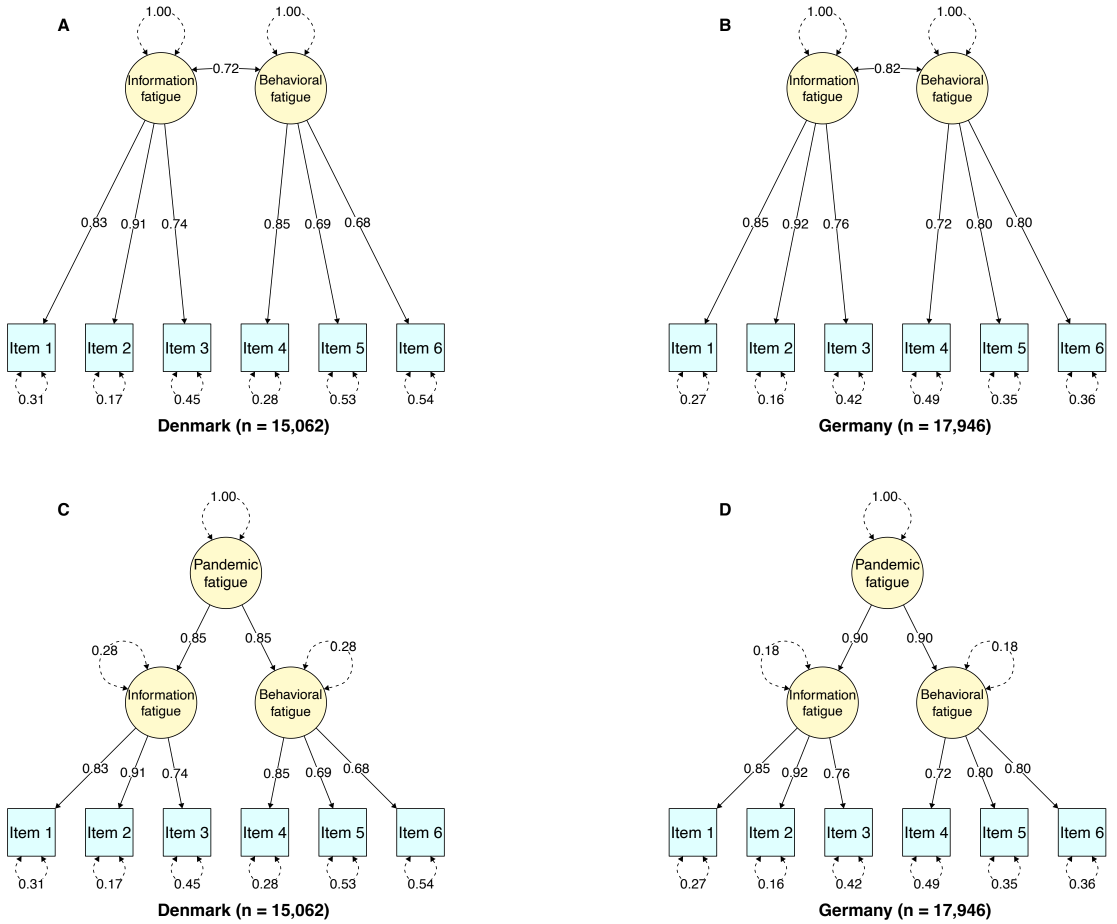

Note. Figure 24A and Figure 24B show the two-factor model of pandemic fatigue with fully standardized factor loadings and (residual) variances for Denmark and Germany, respectively. Figure 24C and Figure 24D show the second-order model of pandemic fatigue with fully standardized factor loadings and (residual) variances for Denmark and Germany, respectively. All models were estimated using robust diagonally weighted least squares estimation[18]. Item 1= 'I am tired of all the COVID-19 discussions in TV shows, newspapers, and radio programs, etc.'; Item 2 = 'I am sick of hearing about COVID-19'; Item 3 = 'When friends or family members talk about COVID-19, I try to change the subject because I do not want to talk about it anymore'; Item 4 = 'I feel strained from following all of the behavioral regulations and recommendations around COVID-19'; Item 5 = 'I am tired of restraining myself to save those who are most vulnerable to COVID-19'; Item 6 = 'I am losing my spirit to fight against COVID-19'. Response scale: 1 = strongly disagree, 2 = disagree, 3 = somewhat disagree, 4 = neutral/neither disagree nor agree, 5 = somewhat agree, 6 = agree, 7 = strongly agree.

Figure S25. Two-factor and second-order models of pandemic fatigue fitted with robust maximum likelihood estimation and robust diagonally weighted least squares estimation in the U.S.

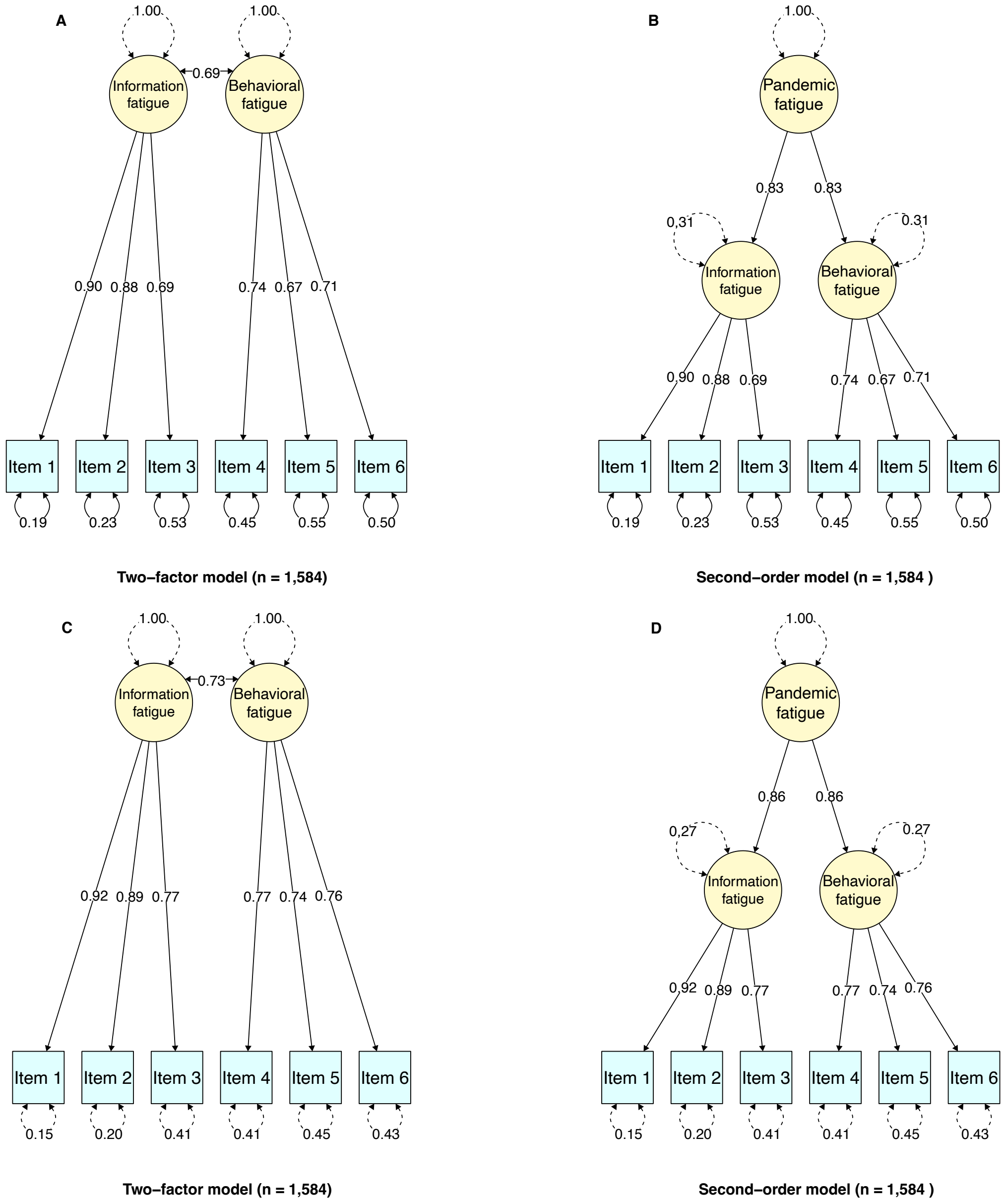

Note. Figure 25A and Figure 25B show the two-factor and second-order model of pandemic fatigue with fully standardized factor loadings and (residual) variances for the U.S. experimental sample fitted using robust maximum likelihood estimation with robust standard errors and a Satorra-Bentler scaled test statistic[23]. Figure 25C and Figure 25D show the two-factor and second-order model of pandemic fatigue with fully standardized factor loadings and (residual) variances for the U.S. experimental sample fitted using robust diagonally weighted least squares estimation[18].Item 1= ‘I am tired of all the COVID-19 discussions in TV shows, newspapers, and radio programs, etc.’; Item 2 = ‘I am sick of hearing about COVID-19’; Item 3 = ‘When friends or family members talk about COVID-19, I try to change the subject because I do not want to talk about it anymore’; Item 4 = ‘I feel strained from following all of the behavioral regulations and recommendations around COVID-19’; Item 5 = ‘I am tired of restraining myself to save those who are most vulnerable to COVID-19’; Item 6 = ‘I am losing my spirit to fight against COVID-19’. Response scale: 1 = strongly disagree, 2 = disagree, 3 = somewhat disagree, 4 = neutral/neither disagree nor agree, 5 = somewhat agree, 6 = agree, 7 = strongly agree.

Figure S26. OLS regressions predicting pandemic fatigue in the U.S.

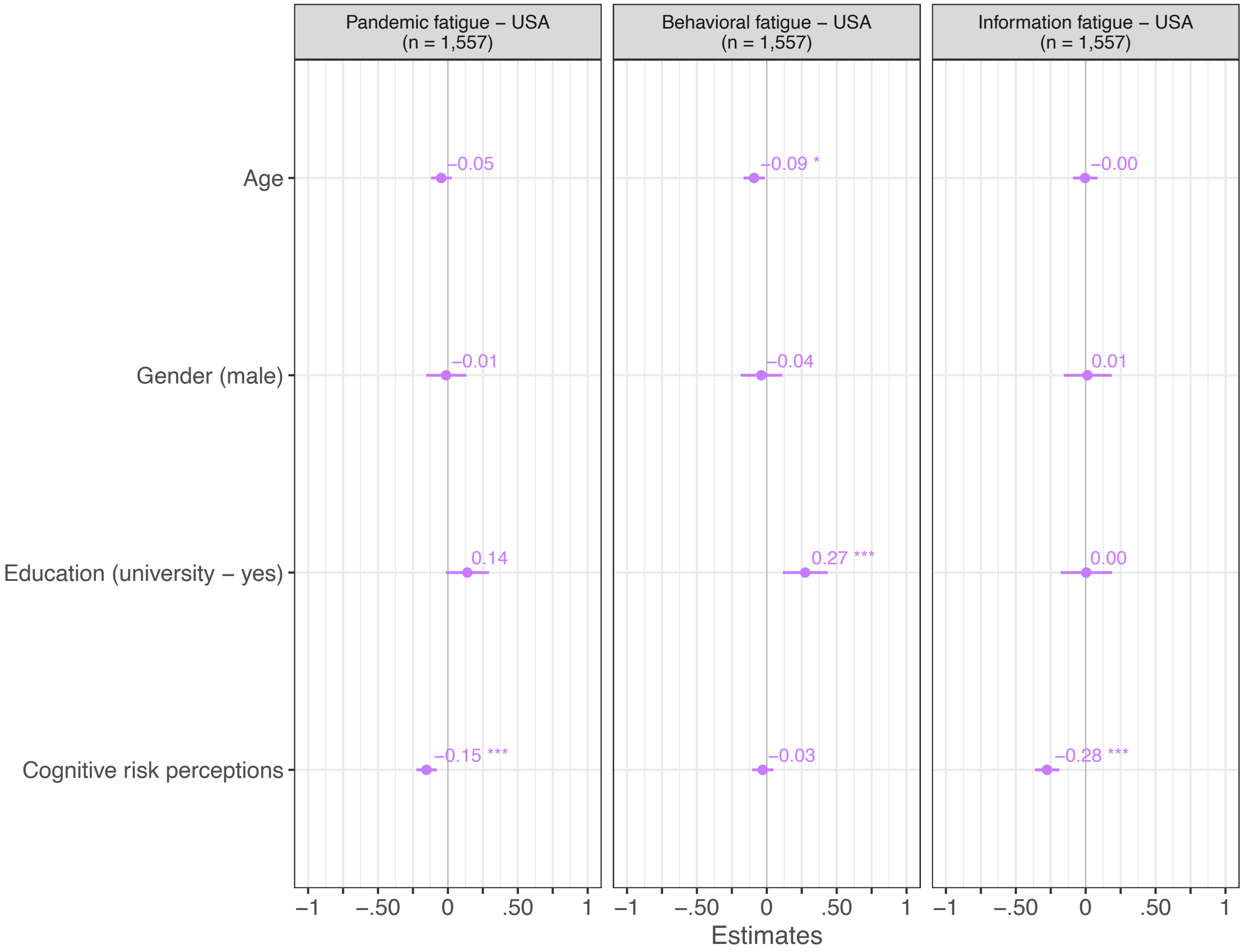

Note. Figure S26 shows standardized β coefficients with 95% confidence intervals based on ordinary least squares regressions with data from the experiment. All continuous predictors have been mean-centered and scaled by 1 standard deviation. The p-values have not been adjusted for multiple comparisons and are presented as follows: $^{***}p_{\text{two-tailed}} < .001$; $^{**}p_{\text{two-tailed}} < 0.01$; $^{*}p_{\text{two-tailed}} < 0.05$. Exact p-values for all models are presented in the R-output which has been deposited on the Open Science Framework at: http://dx.doi.org/10.17605/OSF.IO/XD463. The gender variable refers to participants self-identified gender as presented to them in the experiment. Participants who did not identify as either male or female are not included in the analyses due to an insufficient number of observations.

Table S1. Sociodemographics for the Danish and German repeated cross-sectional survey.

	Danish repeated cross-sectional survey	German repeated cross-sectional survey
**Age**		
Mean	56.54	45.07
SD	15.47	15.72
**Gender**		
Female %	54.60	50.69
Male %	45.18	49.31
Other %	0.22	-
**Education**		
Less than 10 years %	10.81	7.85
10 years or more %	89.19	92.15
**Employment**		
Employed %	52.67	68.12
Unemployed %	47.33	31.88
**Chronic disease status**		
Yes %	29.39	35.48
No %	66.63	61.48
Don’t know %	3.98	3.04
n	15,985	17,946

Note. Standard deviation (SD).

Table S2. Response and completion rate for the Danish repeated cross-sectional survey per wave.

	Invited	Started		Completed		Completed with no technical issues	
Wave	n	n	%	n	%	n	%
19	7,500	1731	23.08	963	12.84	923	12.31
20	7,500	1722	22.96	945	12.60	912	12.16
21	8,000	1823	22.79	993	12.41	957	11.96
22	7,500	1475	19.67	756	10.08	729	9.72
23	7,500	1809	24.12	918	12.24	876	11.68
24	8,500	1870	22.00	1077	12.67	1036	12.19
25	5,250	1370	26.10	738	14.06	715	13.62
26	5,250	1232	23.47	671	12.78	653	12.44
27	5,250	1277	24..32	668	12.72	644	12.27
28	5,250	1290	24.57	735	14.00	718	13.68
29	5,250	1171	22.30	674	12.84	658	12.53
30	5,250	1201	22.88	735	14.00	723	13.77
31	5,250	1030	19.62	624	11.89	606	11.54
32	5,250	964	18.36	576	10.97	557	10.61
33	5,250	870	16.57	486	9.26	475	9.05
34	5,250	890	16.95	536	10.21	521	9.92
35	5,250	930	17.71	512	9.75	504	9.60
36	5,250	853	16.25	493	9.39	489	9.31
37	5,250	791	15.07	453	8.63	444	8.46
38	5,250	723	13.77	444	8.46	438	8.34
39	5,250	818	15.58	482	9.18	473	9.01
40	5,250	856	16.30	490	9.33	483	9.20
41	5,250	792	15.09	468	8.91	465	8.86
42	5,250	804	15.31	490	9.33	485	9.24
43	5,540	806	14.55	511	9.22	501	9.04
Total	146,540	29,098	19.86	16,438	11.22	15,985	10.91

Table S3. Sociodemographics for the Danish panel survey.

	Wave 11	Wave 12	Wave 13	Wave 14	Wave 15	Wave 16	Wave 17	Wave 18	Wave 19
**Age**									
Mean	58.50	58.43	58.46	58.75	59.38	59.39	60.07	59.54	59.47
SD	13.86	14.03	13.89	13.40	13.24	13.43	13.35	13.59	13.08
**Gender**									
Female %	55.71	52.54	54.40	53.45	53.88	53.38	51.57	55.13	55.3
Male %	44.29	47.46	45.60	46.55	46.12	46.62	48.43	44.87	44.7
**Education**									
Less than 10 years %	5.75	3.82	4.64	5.93	5.85	5.53	4.87	3.82	6.03
10 years or more %	94.25	96.18	95.36	94.07	94.15	94.47	95.13	96.18	93.97
**Chronic disease status**									
Yes %	27.59	25.77	26.68	27.48	28.05	28.72	31.90	30.38	29.68
No %	69.43	72.19	70.30	69.80	69.27	68.01	64.66	66.37	66.28
Don’t know %	2.99	2.04	3.02	2.72	2.68	3.27	3.45	3.24	4.03
n	438	394	432	406	412	399	351	341	349

Note. Standard deviation (SD).

Table S4. Means, standard deviations, and Cronbach's α for the Danish repeated cross-sectional survey.

	Number of items	Mean	SD	α
Physical distancing	2	6.28	1.03	0.64
Hygiene	3	6.16	0.87	0.63
Mask wearing	1	2.22	1.31	-
Information seeking	5	5.34	1.28	0.88
Pandemic fatigue	6	3.36	1.33	0.83
Behavioral fatigue	3	2.87	1.40	0.73
Information fatigue	3	3.85	1.61	0.83
Cognitive risk perceptions	2	13.41	9.00	-
Affective risk perceptions	6	4.39	1.02	0.78
Institutional trust	6	5.35	0.98	0.83
Worries – personal/societal	7	4.02	1.09	0.74
Optimism about the future	1	3.66	0.91	-
Negative affect	4	2.24	1.02	0.83
Empathy – most vulnerable	3	5.64	1.24	0.86
Honesty-Humility	4	4.36	0.58	0.38
Emotionality	4	2.92	0.67	0.32
Extraversion	4	4.13	0.68	0.63
Agreeableness vs. anger	4	3.36	0.66	0.46
Conscientiousness	4	3.79	0.66	0.50
Openness to experience	4	3.63	0.71	0.54

Note. Standard deviation (SD). Participants' cognitive risk perception was estimated by taking the product of one item measuring participants' assessment of how serious it would be for them to get infected and one item asking them to judge their own likelihood of contracting the virus.

Table S5. Means, standard deviations, and Cronbach's α for the German repeated cross-sectional survey.

	Number of items	Mean	SD	α
Physical distancing	10	4.24	0.73	0.91
Hygiene	5	4.16	0.70	0.75
Mask wearing	1	4.58	0.76	-
Information seeking	1	5.25	1.50	-
Pandemic fatigue	6	3.65	1.54	0.86
Behavioral fatigue	3	3.14	1.62	0.77
Information fatigue	3	4.16	1.77	0.84
Cognitive risk perceptions	2	1.50	9.20	-
Affective risk perceptions	6	4.55	1.15	0.80
Institutional trust	11	4.26	1.41	0.94
Worries – personal/societal	9	4.88	1.04	0.76

Note. Standard deviation (SD). Participants' cognitive risk perception was estimated by taking the product of one item measuring participants' assessment of how serious it would be for them to get infected and one item asking them to judge their own likelihood of contracting the virus.

Table S6. Means, standard deviations, and Cronbach's α for each wave of the Danish panel survey.

	Wave 11	Wave 12	Wave 13	Wave 14	Wave 15	Wave 16	Wave 17	Wave 18	Wave 19
**Physical distancing**									
Number of items	2	2	2	2	2	2	2	2	2
Mean	6.52	6.54	6.63	6.70	6.60	6.54	6.45	6.23	5.95
SD	0.78	0.70	0.66	0.55	0.61	0.67	0.70	0.92	1.07
α	0.70	0.60	0.63	0.54	0.51	0.60	0.57	0.64	0.68
**Hygiene**									
Number of items	3	3	3	3	3	3	3	3	3
Mean	6.23	6.24	6.29	6.24	6.19	6.13	6.02	5.97	5.91
SD	0.84	0.82	0.73	0.78	0.83	0.83	0.94	0.90	0.92
α	0.68	0.66	0.56	0.57	0.62	0.61	0.70	0.64	0.63
**Mask wearing**									
Number of items	1	1	1	1	1	1	1	1	1
Mean	1.87	2.13	2.29	2.17	2.02	2.25	2.24	2.24	1.89
SD	0.98	1.24	1.30	1.27	1.18	1.35	1.30	1.31	1.09
α	-	-	-	-	-	-	-	-	-
**Information seeking**									
Number of items	-	5	5	5	5	5	5	5	5
Mean	-	5.16	5.41	5.34	5.18	5.19	5.12	4.85	4.89
SD	-	1.24	1.20	1.17	1.27	1.24	1.26	1.34	1.23
A	-	0.86	0.86	0.86	0.87	0.88	0.89	0.89	0.85
**Pandemic fatigue**									
Number of items	6	6	6	6	6	6	6	6	6
Mean	2.81	3.12	3.07	3.20	3.52	3.49	3.39	3.36	3.06
SD	1.30	1.25	1.37	1.41	1.44	1.32	1.33	1.34	1.36
α	0.86	0.84	0.86	0.87	0.87	0.85	0.86	0.86	0.87
**Behavioral fatigue**									
Number of items	3	3	3	3	3	3	3	3	3
Mean	2.37	2.63	2.71	2.83	3.10	3.00	2.86	2.79	2.44
SD	1.29	1.25	1.44	1.46	1.51	1.42	1.34	1.40	1.30
α	0.80	0.71	0.79	0.78	0.80	0.76	0.75	0.76	0.77

Table S6. Continued.

	Wave 11	Wave 12	Wave 13	Wave 14	Wave 15	Wave 16	Wave 17	Wave 18	Wave 19
**Information fatigue**									
Number of items	3	3	3	3	3	3	3	3	3
Mean	3.25	3.60	3.43	3.56	3.93	3.98	3.91	3.93	3.68
SD	1.62	1.55	1.64	1.66	1.67	1.54	1.63	1.59	1.73
α	0.85	0.84	0.86	0.87	0.87	0.86	0.88	0.86	0.89
**Cognitive risk perceptions**									
Number of items	2	2	2	2	2	2	2	2	2
Mean	17.18	17.27	18.32	16.45	15.40	14.19	12.30	9.17	9.38
SD	9.37	8.93	9.86	8.83	9.36	8.45	7.62	6.98	7.34
α	-	-	-	-	-	-	-	-	-
**Affective risk perceptions**									
Number of items	6	6	6	6	6	6	6	6	6
Mean	4.54	4.59	4.92	4.73	4.66	4.54	4.41	4.22	4.22
SD	0.98	0.92	0.96	0.91	0.98	0.94	0.96	0.98	1.01
α	0.78	0.76	0.79	0.76	0.78	0.76	0.76	0.76	0.79
**Institutional trust**									
Number of items	6	6	6	6	6	6	6	6	6
Mean	5.44	5.38	5.52	5.61	5.45	5.53	5.56	5.56	5.57
SD	0.98	0.94	0.96	0.93	0.95	0.92	0.95	0.94	0.89
α	0.87	0.84	0.85	0.86	0.84	0.83	0.83	0.83	0.82
**Worries – personal/societal**									
Number of items	7	7	7	7	7	7	7	7	7
Mean	3.82	3.82	3.99	3.94	3.93	3.87	3.72	3.42	3.32
SD	1.06	1.04	0.98	0.97	1.01	1.05	1.13	1.20	1.16
α	0.74	0.75	0.72	0.73	0.73	0.77	0.80	0.82	0.80

Table S6. Continued.

	Wave 11	Wave 12	Wave 13	Wave 14	Wave 15	Wave 16	Wave 17	Wave 18	Wave 19
**Optimism about the future**									
Number of items	1	1	1	1	1	1	1	1	1
Mean	3.52	3.47	3.57	3.55	3.48	3.60	3.64	3.69	3.78
SD	0.87	0.84	0.89	0.90	0.86	0.87	0.83	0.88	0.82
α	-	-	-	-	-	-	-	-	-
**Negative affect**									
Number of items	4	4	4	4	4	4	4	4	4
Mean	1.91	2.03	2.07	2.31	2.39	2.27	2.06	1.85	1.73
SD	0.93	0.99	1.00	1.08	1.12	1.11	1.07	0.97	0.87
α	0.85	0.86	0.83	0.86	0.87	0.88	0.89	0.88	0.87
**Empathy – most vulnerable**									
Number of items	3	3	3	3	3	3	3	3	3
Mean	5.59	5.62	5.76	5.74	5.60	5.65	5.50	5.34	5.43
SD	1.29	1.17	1.22	1.19	1.28	1.14	1.23	1.29	1.23
α	0.89	0.89	0.90	0.90	0.90	0.88	0.89	0.88	0.89
**Honesty-Humility**									
Number of items	4	4	4	4	4	4	4	4	4
Mean	4.40	4.39	4.36	4.40	4.38	4.39	4.35	4.39	4.37
SD	0.53	0.55	0.55	0.53	0.56	0.54	0.56	0.55	0.56
α	0.35	0.38	0.32	0.35	0.37	0.34	0.37	0.35	0.34
**Emotionality**									
Number of items	4	4	4	4	4	4	4	4	4
Mean	2.93	2.87	2.88	2.86	2.88	2.90	2.87	2.87	2.92
SD	0.63	0.66	0.64	0.63	0.65	0.62	0.65	0.64	0.65
α	0.25	0.30	0.25	0.24	0.28	0.22	0.28	0.25	0.30

Table S6. Continued.

	Wave 11	Wave 12	Wave 13	Wave 14	Wave 15	Wave 16	Wave 17	Wave 18	Wave 19
**Extraversion**									
Number of items	4	4	4	4	4	4	4	4	4
Mean	4.07	4.11	4.11	4.13	4.13	4.10	4.10	4.08	4.09
SD	0.69	0.67	0.69	0.67	0.66	0.67	0.67	0.70	0.67
α	0.67	0.64	0.64	0.63	0.63	0.61	0.61	0.65	0.61
**Agreeableness vs. anger**									
Number of items	4	4	4	4	4	4	4	4	4
Mean	3.38	3.38	3.35	3.34	3.34	3.35	3.33	3.36	3.36
SD	0.65	0.64	0.66	0.67	0.63	0.66	0.68	0.66	0.67
α	0.54	0.48	0.55	0.55	0.49	0.54	0.55	0.51	0.54
**Conscientiousness**									
Number of items	4	4	4	4	4	4	4	4	4
Mean	3.85	3.86	3.85	3.85	3.82	3.81	3.87	3.85	3.85
SD	0.59	0.58	0.58	0.59	0.59	0.59	0.59	0.58	0.57
α	0.41	0.37	0.36	0.40	0.39	0.41	0.40	0.41	0.34
**Openness to experience**									
Number of items	4	4	4	4	4	4	4	4	4
Mean	3.67	3.72	3.71	3.69	3.67	3.71	3.73	3.70	3.71
SD	0.69	0.67	0.70	0.70	0.71	0.70	0.67	0.69	0.70
α	0.54	0.49	0.53	0.52	0.54	0.53	0.49	0.51	0.50

Note. Standard deviation (SD). Participants' cognitive risk perception was estimated by taking the product of one item measuring participants' assessment of how serious it would be for them to get infected and one item asking them to judge their own likelihood of contracting the virus.

Table S7. Item overview Danish repeated cross-sectional survey.

Variable	Item	Response format
Age		
	What year were you born?	1900-2020
Gender		
	What is your gender?	(1) Male<br><br>(2) Female<br><br>(3) None of the above
Education		
	What is your highest level of education completed?	(1) No schooling, pre-school, or primary school (Danish 1st-5th grade)<br><br>(2) Middle school (Danish 6th-8th grades)<br><br>(3) Middle/high school (Danish 9th-10th grade)<br><br>(4) High school (Danish 12th grade completed)<br><br>(5) Short vocational education, basic programme completed<br><br>(6) Vocational education, main programme completed

		(7) Middle-length higher education of 2-3 years<br><br>(8) Middle-length higher education of 3-4 years<br><br>(9) University degree, bachelor level<br>(10) University degree, master's level<br><br>(11) Licentiate<br><br>(12) Ph.D.<br><br>(99) Other
Employment	What best describes your current situation?	(1) I am a student<br><br>(2) I have a job<br><br>(3) I have my own company<br><br>(4) I am unemployed<br><br>(5) I am retired<br><br>(6) I am retired early and/or receive incapacity benefit<br><br>(99) Other

Chronic disease status	Do you suffer from any chronic illness?	(1) Yes<br>(2) No<br>(99) Don't know
Pandemic fatigue	Please indicate the extent to which you disagree or agree with the following statements.<br>I am tired of all the COVID-19 discussions in TV shows, newspapers, and radio programs, etc.<br>I am sick of hearing about COVID-19<br>When friends or family members talk about COVID-19, I try to change the subject because I do not want to talk about it anymore<br>I feel strained from following all of the behavioural regulations and recommendations around COVID-19<br>I am tired of restraining myself to save those who are most vulnerable to COVID-19<br>I am losing my spirit to fight against COVID-19	(1) Strongly disagree<br>-<br>(7) Strongly agree
Cognitive risk perceptions	How likely do you think it is that you will be infected with the novel coronavirus (COVID-19)?	(1) Very unlikely<br>-<br>(7) Very likely
	How serious would it be for you if you contracted the novel coronavirus (COVID-19)?	(1) Not serious at all<br>-<br>(7) Very serious

Affective risk perceptions	To me, the novel coronavirus (COVID-19) feels...	(1) Close to me - (7) Far away from me
	To me, the novel coronavirus (COVID-19) feels...	(1) Terrifying - (7) Not terrifying
	To me, the novel coronavirus (COVID-19) feels...	(1) Like something that makes me feel helpless - (7) Like something I can combat with my own actions
	To me, the novel coronavirus (COVID-19) feels...	(1) Slowly spreading - (7) Fast spreading
	To me, the novel coronavirus (COVID-19) feels...	(1) Like something I think of all the time - (7) Like something I don't think about at all
	To me, the novel coronavirus (COVID-19) feels...	(1) Like something to worry about - (7) Like something not to worry about

Institutional trust	How much confidence do you have that the following individuals and organizations are capable of handling the novel coronavirus well and correctly?<br><br>The police<br><br>Private businesses<br><br>Hospitals and doctors<br><br>State authorities<br><br>Experts (e.g., researchers)<br><br>Politicians	(1) Very low confidence<br>-<br>(7) Very high confidence
Worries – personal/societal	Crises often involve fears and worries. At present, how much do you worry about:<br><br>Loosing someone I love<br><br>The health care system being overloaded<br><br>Small businesses going bankrupt<br><br>Economic recession<br><br>Shortage of food supplies<br><br>Blackouts<br><br>Society becoming more egoistic	(1) Don´t worry me at all<br>-<br>(7) Worries me a lot
Physical distancing	Please indicate the extent to which you disagree or agree with the following statements.<br><br>I keep a distance to the elderly and/or people that I know to suffer from a chronic illness<br><br>I try to limit the amount of physical contact I have with others (e.g., handshakes, kisses on the cheek, hugs)	(1) Strongly disagree<br>-<br>(7) Strongly agree

Hygiene	Please indicate the extent to which you disagree or agree with the following statements.<br><br>I wash my hands often or use hand disinfectant<br><br>I pay extra attention to cleaning at the moment<br><br>I make sure to cough or sneeze in my sleeve rather than in my hands	(1) Strongly disagree<br>-<br>(7) Strongly agree
Mask wearing	Do you use a mask when you go outside?	(1) Never<br>-<br>(5) Always
Information seeking	Please indicate the extent to which you disagree or agree with the following statements.<br><br>I regularly seek out information on the current COVID-19 situation.<br><br>I try to stay updated on the current COVID-19 restrictions.<br><br>I often read, listen to, or watch news about COVID-19.<br><br>I closely follow the announcements from the government and/or the health authorities concerning COVID-19.<br><br>I spend a considerable amount of time learning more about COVID-19.	(1) Strongly disagree<br>-<br>(7) Strongly agree
Negative affect	Please answer the following questions.<br><br>I am bored at the moment<br><br>I feel lonely at the moment<br><br>I feel isolated at the moment<br><br>I feel stressed out at the moment	(1) Not at all<br>-<br>(5) Extremely

Optimism about the future	Please answer the following question.<br><br>I am very optimistic when I think about the future	(1) Not at all<br>-<br>(5) Extremely
Empathy – most vulnerable	Please indicate the extent to which you disagree or agree with the following statements.<br><br>I am very concerned about those most vulnerable to the novel coronavirus (COVID-19)<br><br>I feel compassion for those most vulnerable to the novel coronavirus (COVID-19)<br><br>I am quite affected by what could happen to those most vulnerable to the novel coronavirus (COVID-19)	(1) Strongly disagree<br>-<br>(7) Strongly agree
Honesty-Humility	Please read the statements listed below and indicate for each of these to which extent you agree or disagree. Note that there are no "right" or "wrong" answers. Please answer all statements even if you aren't completely certain of your answer. If nothing else is specified, the statements refer to your behaviour (towards other people) or your general opinion.<br><br>I find it difficult to lie<br><br>I would like to know how to make lots of money in a dishonest manner<br><br>I want to be famous<br><br>I am entitled to special treatment	(1) Strongly disagree<br>-<br>(5) Strongly agree

Emotionality	Please read the statements listed below and indicate for each of these to which extent you agree or disagree. Note that there are no "right" or "wrong" answers. Please answer all statements even if you aren't completely certain of your answer. If nothing else is specified, the statements refer to your behaviour (towards other people) or your general opinion.<br><br>I am afraid of feeling pain<br><br>I worry less than others<br><br>I can easily overcome difficulties on my own<br><br>I have to cry during sad or romantic movies	(1) Strongly disagree<br>-<br>(5) Strongly agree
Extraversion	Please read the statements listed below and indicate for each of these to which extent you agree or disagree. Note that there are no "right" or "wrong" answers. Please answer all statements even if you aren't completely certain of your answer. If nothing else is specified, the statements refer to your behaviour (towards other people) or your general opinion.<br><br>Nobody likes talking with me<br><br>I easily approach strangers<br><br>I like to talk with others<br><br>I am seldom cheerful	(1) Strongly disagree<br>-<br>(5) Strongly agree

Agreeableness vs. Anger	Please read the statements listed below and indicate for each of these to which extent you agree or disagree. Note that there are no "right" or "wrong" answers. Please answer all statements even if you aren't completely certain of your answer. If nothing else is specified, the statements refer to your behaviour (towards other people) or your general opinion.<br><br>I remain unfriendly to someone who was mean to me<br><br>I often express criticism<br><br>I tend to quickly agree with others<br><br>Even when I'm treated badly, I remain calm	(1) Strongly disagree<br>-<br>(5) Strongly agree
Conscientiousness	Please read the statements listed below and indicate for each of these to which extent you agree or disagree. Note that there are no "right" or "wrong" answers. Please answer all statements even if you aren't completely certain of your answer. If nothing else is specified, the statements refer to your behaviour (towards other people) or your general opinion.<br><br>I make sure that things are in the right spot<br><br>I postpone complicated tasks as long as possible<br><br>I work very precisely<br><br>I often do things without really thinking	(1) Strongly disagree<br>-<br>(5) Strongly agree

Openness to Experience	Please read the statements listed below and indicate for each of these to which extent you agree or disagree. Note that there are no "right" or "wrong" answers. Please answer all statements even if you aren't completely certain of your answer. If nothing else is specified, the statements refer to your behaviour (towards other people) or your general opinion.<br><br>I can look at a painting for a long time<br><br>I think science is boring<br><br>I have a lot of imagination<br><br>I like people with strange ideas	(1) Strongly disagree<br>-<br>(5) Strongly agree

Table S8. Item overview German repeated cross-sectional survey.

Variable	Item	Response format
Age	How old are you?	0-100 years
Gender	What is your gender?	(1) Male<br>(2) Female
Education	Please provide information about your education.	(1) Up to 9 years of schooling<br>(2) At least 10 years (without university qualification)<br>(3) At least 10 years (with university qualification)
Employment	Are you employed?	(1) Yes<br>(2) No
Chronic disease status	Do you suffer from any chronic illness?	(1) Yes<br>(2) No<br>(99) Don’t know

Pandemic fatigue	Please indicate the extent to which you disagree or agree with the following statements.	
	I am tired of all the COVID-19 discussions in TV shows, newspapers, and radio programs, etc.<br><br>I am sick of hearing about COVID-19<br><br>When friends or family members talk about COVID-19, I try to change the subject because I do not want to talk about it anymore<br><br>I feel strained from following all of the behavioural regulations and recommendations around COVID-19<br><br>I am tired of restraining myself to save those who are most vulnerable to COVID-19<br><br>I am losing my spirit to fight against COVID-19	(1) Strongly disagree<br>-<br>(7) Strongly agree
Cognitive risk perceptions		
	How likely do you think it is that you will be infected with the novel coronavirus (COVID-19)?	(1) Very unlikely<br>-<br>(7) Very likely
	How serious would it be for you if you contracted the novel coronavirus (COVID-19)?	(1) Not serious at all<br>-<br>(7) Very serious

Affective risk perceptions		
	To me, the novel coronavirus (COVID-19) feels...	(1) Close to me<br>-<br>(7) Far away from me
	To me, the novel coronavirus (COVID-19) feels...	(1) Terrifying<br>-<br>(7) Not terrifying
	To me, the novel coronavirus (COVID-19) feels...	(1) Like something that makes me feel helpless<br>-<br>(7) Like something I can combat with my own actions
	To me, the novel coronavirus (COVID-19) feels...	(1) Slowly spreading<br>-<br>(7) Fast spreading
	To me, the novel coronavirus (COVID-19) feels...	(1) Like something I think of all the time<br>-<br>(7) Like something I don't think about at all
	To me, the novel coronavirus (COVID-19) feels...	(1) Like something to worry about<br>-<br>(7) Like something not to worry about

Institutional trust	How much confidence do you have that the following individuals and organizations are capable of handling the novel coronavirus well and correctly?<br><br>Media<br><br>Hospitals<br><br>Your doctor<br><br>Local health authorities<br><br>Ministry of Health in your state<br><br>Federal Ministry of Health<br><br>Robert Koch Institute (RKI)<br><br>Federal Centre for Health Education (BZgA)<br><br>Science<br><br>Federal government<br><br>World Health Organization (WHO)	(1) Very low confidence<br>-<br>(7) Very high confidence

Worries – personal/societal	Crises often involve fears and worries. At present, how much do you worry about:<br><br>Loosing someone I love<br><br>The health care system being overloaded<br><br>Small businesses going bankrupt<br><br>Economic recession<br><br>Society becoming more egoistic<br><br>Financial difficulties due to a loss of income<br><br>An increasing gap between the rich and the poor<br><br>Getting sick<br><br>Restrictions on social life in the long run	(1) Don´t worry me at all<br>-<br>(7) Worries me a lot

Physical distancing	During the past week how often have you adhered to the following regulations to avoid the spread and infection of the novel coronavirus?	
	Avoided handshakes	
	Kept 1.5 meters distance to other people in public places	
	Avoided public places	
	Avoided private parties	
	Only gone out to do what is necessary (e.g., grocery shopping)	(1) Never - (5) Always
	Refrained from travelling	
	Avoided crowded rooms with poor ventilation	
	Avoided crowded places	
	Avoided lively conversations and close contact with others	
	Avoided people I know	
Hygiene	During the past week how often have you adhered to the following regulations to avoid the spread and infection of the novel coronavirus?	
	Avoided touching my eyes, nose, and mouth with unwashed hands	
	Used hand sanitizer	
	Covered my mouth when coughing	(1) Never - (5) Always
	Washed my hands for 20 seconds	
	Ventilated rooms on a regular basis	

Mask wearing	During the past week how often have you adhered to the following regulations to avoid the spread and infection of the novel coronavirus?<br><br>Worn a mask	(1) Never<br>-<br>(5) Always
Information seeking	How often do you seek out information about the Coronavirus / COVID-19?	(1) Never<br>-<br>(7) Very often

Table S9. Item overview Danish panel survey.

Variable	Item	Response format
Age		
	What year were you born?	1900-2020
Gender		
	What is your gender?	(0) Male<br><br>(1) Female
Education		
	What is your highest level of education completed?	(1) No schooling, pre-school, or primary school (Danish 1st-5th grade)<br><br>(2) Middle school (Danish 6th-8th grades)<br><br>(3) Middle/high school (Danish 9th-10th grade)<br><br>(4) High school (Danish 12th grade completed)<br><br>(5) Short vocational education, basic programme completed<br><br>(6) Vocational education, main programme completed<br><br>(7) Middle-length higher education of 2-3 years

		(8) Middle-length higher education of 3-4 years<br><br>(9) University degree, bachelor level<br>(10) University degree, master's level<br><br>(11) Licentiate<br><br>(12) Ph.D.<br><br>(99) Other
Chronic disease status	Do you suffer from any chronic illness?	(1) Yes<br><br>(2) No<br><br>(99) Don't know
Pandemic fatigue	Please indicate the extent to which you disagree or agree with the following statements.<br>I am tired of all the COVID-19 discussions in TV shows, newspapers, and radio programs, etc.<br><br>I am sick of hearing about COVID-19<br><br>When friends or family members talk about COVID-19, I try to change the subject because I do not want to talk about it anymore<br><br>I feel strained from following all of the behavioural regulations and recommendations around COVID-19<br><br>I am tired of restraining myself to save those who are most vulnerable to COVID-19<br><br>I am losing my spirit to fight against COVID-19	(1) Strongly disagree<br>-<br>(7) Strongly agree

Cognitive risk perceptions	How likely do you think it is that you will be infected with the novel coronavirus (COVID-19)?	(1) Very unlikely<br>-<br>(7) Very likely
	How serious would it be for you if you contracted the novel coronavirus (COVID-19)?	(1) Not serious at all<br>-<br>(7) Very serious
Affective risk perceptions	To me, the novel coronavirus (COVID-19) feels...	(1) Close to me<br>-<br>(7) Far away from me
	To me, the novel coronavirus (COVID-19) feels...	(1) Terrifying<br>-<br>(7) Not terrifying
	To me, the novel coronavirus (COVID-19) feels...	(1) Like something that makes me feel helpless<br>-<br>(7) Like something I can combat with my own actions
	To me, the novel coronavirus (COVID-19) feels...	(1) Slowly spreading<br>-<br>(7) Fast spreading

	To me, the novel coronavirus (COVID-19) feels...	(1) Like something I think of all the time<br>-<br>(7) Like something I don't think about at all
	To me, the novel coronavirus (COVID-19) feels...	(1) Like something to worry about<br>-<br>(7) Like something not to worry about
Institutional trust	How much confidence do you have that the following individuals and organizations are capable of handling the novel coronavirus well and correctly?<br><br>The police<br><br>Private businesses<br><br>Hospitals and doctors<br><br>State authorities<br><br>Experts (e.g., researchers)<br><br>Politician	(1) Very low confidence<br>-<br>(7) Very high confidence

Worries – personal/societal	Crises often involve fears and worries. At present, how much do you worry about:<br><br>Loosing someone I love<br><br>The health care system being overloaded<br><br>Small businesses going bankrupt<br><br>Economic recession<br><br>Shortage of food supplies<br><br>Blackouts<br><br>Society becoming more egoistic	(1) Don´t worry me at all<br>-<br>(7) Worries me a lot
Physical distancing	Please indicate the extent to which you disagree or agree with the following statements.<br><br>I keep a distance to the elderly and/or people that I know to suffer from a chronic illness<br><br>I try to limit the amount of physical contact I have with others (e.g., handshakes, kisses on the cheek, hugs)	(1) Strongly disagree<br>-<br>(7) Strongly agree
Hygiene	Please indicate the extent to which you disagree or agree with the following statements.<br><br>I wash my hands often or use hand disinfectant<br><br>I pay extra attention to cleaning at the moment<br><br>I make sure to cough or sneeze in my sleeve rather than in my hands	(1) Strongly disagree<br>-<br>(7) Strongly agree
Mask wearing	Do you use a mask when you go outside?	(1) Never<br>-<br>(5) Always

Information seeking	Please indicate the extent to which you disagree or agree with the following statements.<br><br>I regularly seek out information on the current COVID-19 situation.<br><br>I try to stay updated on the current COVID-19 restrictions.<br><br>I often read, listen to, or watch news about COVID-19.<br><br>I closely follow the announcements from the government and/or the health authorities concerning COVID-19.<br><br>I spend a considerable amount of time learning more about COVID-19.	(1) Strongly disagree<br>-<br>(7) Strongly agree
Negative affect	Please answer the following questions.<br><br>I am bored at the moment<br><br>I feel lonely at the moment<br><br>I feel isolated at the moment<br><br>I feel stressed out at the moment	(1) Not at all<br>-<br>(5) Extremely
Optimism about the future	Please answer the following question.<br><br>I am very optimistic when I think about the future	(1) Not at all<br>-<br>(5) Extremely

Empathy – most vulnerable	Please indicate the extent to which you disagree or agree with the following statements.<br><br>I am very concerned about those most vulnerable to the novel coronavirus (COVID-19)<br><br>I feel compassion for those most vulnerable to the novel coronavirus (COVID-19)<br><br>I am quite affected by what could happen to those most vulnerable to the novel coronavirus (COVID-19)	(1) Strongly disagree<br>-<br>(7) Strongly agree
Honesty-Humility	Please read the statements listed below and indicate for each of these to which extent you agree or disagree. Note that there are no "right" or "wrong" answers. Please answer all statements even if you aren't completely certain of your answer. If nothing else is specified, the statements refer to your behaviour (towards other people) or your general opinion.<br><br>I find it difficult to lie<br><br>I would like to know how to make lots of money in a dishonest manner<br><br>I want to be famous<br><br>I am entitled to special treatment	(1) Strongly disagree<br>-<br>(5) Strongly agree

Emotionality	Please read the statements listed below and indicate for each of these to which extent you agree or disagree. Note that there are no "right" or "wrong" answers. Please answer all statements even if you aren't completely certain of your answer. If nothing else is specified, the statements refer to your behaviour (towards other people) or your general opinion.<br><br>I am afraid of feeling pain<br><br>I worry less than others<br><br>I can easily overcome difficulties on my own<br><br>I have to cry during sad or romantic movies	(1) Strongly disagree<br>-<br>(5) Strongly agree
Extraversion	Please read the statements listed below and indicate for each of these to which extent you agree or disagree. Note that there are no "right" or "wrong" answers. Please answer all statements even if you aren't completely certain of your answer. If nothing else is specified, the statements refer to your behaviour (towards other people) or your general opinion.<br><br>Nobody likes talking with me<br><br>I easily approach strangers<br><br>I like to talk with others<br><br>I am seldom cheerful	(1) Strongly disagree<br>-<br>(5) Strongly agree

Agreeableness vs. Anger	Please read the statements listed below and indicate for each of these to which extent you agree or disagree. Note that there are no "right" or "wrong" answers. Please answer all statements even if you aren't completely certain of your answer. If nothing else is specified, the statements refer to your behaviour (towards other people) or your general opinion.<br><br>I remain unfriendly to someone who was mean to me<br><br>I often express criticism<br><br>I tend to quickly agree with others<br><br>Even when I'm treated badly, I remain calm	(1) Strongly disagree<br>-<br>(5) Strongly agree
Conscientiousness	Please read the statements listed below and indicate for each of these to which extent you agree or disagree. Note that there are no "right" or "wrong" answers. Please answer all statements even if you aren't completely certain of your answer. If nothing else is specified, the statements refer to your behaviour (towards other people) or your general opinion.<br><br>I make sure that things are in the right spot<br><br>I postpone complicated tasks as long as possible<br><br>I work very precisely<br><br>I often do things without really thinking	(1) Strongly disagree<br>-<br>(5) Strongly agree

Openness to Experience	Please read the statements listed below and indicate for each of these to which extent you agree or disagree. Note that there are no "right" or "wrong" answers. Please answer all statements even if you aren't completely certain of your answer. If nothing else is specified, the statements refer to your behaviour (towards other people) or your general opinion.<br><br>I can look at a painting for a long time<br><br>I think science is boring<br><br>I have a lot of imagination<br><br>I like people with strange ideas	(1) Strongly disagree<br>-<br>(5) Strongly agree

Table S10. The initial 10-item pandemic fatigue scale.

1. It bothers me to adhere to the behavioral guidelines.
2. I am tired of restricting my liberty to avoid the spread of COVID-19.
3. I am tired of all the COVID-19 discussions in talk shows, the radio etc.
4. I am exhausted from trying to keep up with the constantly changing recommendations around COVID-19.
5. I feel strained from following all of the behavioral regulations and guidelines with regard to COVID-19.
6. I am sick of hearing about COVID-19.
7. I am tired of restraining myself to save those who are particularly vulnerable to COVID-19.
8. These days I am finding it more and more difficult to force myself to follow the COVID-19 regulations.
9. When friends or family members talk about COVID-19, I try to change the subject because I don't want to talk about it anymore.
10. I am losing my spirit to fight against COVID-19.

Note. Response scale: 1 = strongly disagree, 2 = disagree, 3 = somewhat disagree, 4 = neutral/neither disagree nor agree, 5 = somewhat agree, 6 = agree, 7 = strongly agree.

Table S11. Very simple structure and Velicer's minimum average partial by number of factors based on Pearson product-moment correlations.

Factors	VVS1	VVS2	MAP
1	.89	.00	.03
2	.66	.74	.03
3	.41	.61	.06
4	.41	.52	.09
5	.33	.46	.14

Note. Very simple structure (VVS); minimum average partial (MAP).

Table S12. Robust fit statistics for the second-order model across waves in the Danish repeated cross-sectional survey based on robust maximum likelihood estimation.

Wave	n	CFI	TLI	RMSEA	SRMR
20	912	.991	.983	.050	.026
21	957	.980	.963	.072	.035
22	729	.977	.957	.079	.038
23	876	.990	.981	.051	.028
24	1,036	.984	.970	.066	.033
25	715	.988	.978	.059	.029
26	653	.988	.978	.055	.030
27	644	.990	.981	.056	.023
28	718	.991	.983	.051	.030
29	658	.976	.955	.080	.037
30	723	.986	.973	.066	.036
31	606	.983	.968	.067	.037
32	557	.965	.934	.096	.052
33	475	.964	.932	.104	.051
34	521	.982	.966	.075	.032
35	504	.992	.985	.047	.027
36	489	.987	.975	.061	.033
37	444	.974	.951	.084	.043
38	438	.987	.975	.066	.031
39	473	.996	.993	.036	.017
40	483	.986	.973	.061	.036
41	465	.997	.995	.027	.028
42	485	.982	.966	.073	.038
43	501	.995	.991	.037	.028

Table S13. Robust fit statistics for the second-order model across waves in the German repeated cross-sectional survey based on robust maximum likelihood estimation.

Wave	n	CFI	TLI	RMSEA	SRMR
24	1,018	.998	.995	.030	.017
25	1,013	.985	.971	.075	.032
26	1,018	.987	.976	.070	.029
31	973	.983	.968	.071	.032
32	1,007	.988	.977	.064	.032
33	1,014	.977	.958	.084	.034
34	1,001	.988	.978	.063	.025
35	1,021	982	.967	.075	.034
37	1,012	.984	.970	.067	.031
38	994	.987	.975	.067	.030
39	1,014	.997	.995	.028	.019
40	976	.991	.984	.052	.028
41	997	.988	.978	.058	.026
42	1,040	.975	.953	.086	.038
43	950	.988	.977	.064	.026
44	980	.988	.977	.061	.028
49	967	.983	.969	.076	.029
51	951	.991	.983	.056	.027

Table S14. Robust fit statistics for the second-order model across waves in the Danish repeated cross-sectional survey based on robust diagonally weighted least squares estimation.

Wave	n	CFI	TLI	RMSEA	SRMR
20	912	.990	.982	.087	.028
21	957	.985	.971	.105	.035
22	729	.983	.968	.114	.037
23	876	.990	.982	.083	.028
24	1,036	.985	.971	.107	.035
25	715	.993	.988	.080	.028
26	653	.992	.985	.080	.030
27	644	.996	.992	.065	.023
28	718	.991	.983	.088	.029
29	658	.984	.969	.107	.035
30	723	.990	.982	.097	.032
31	606	.988	.978	.089	.032
32	557	.970	.944	.148	.051
33	475	.975	.953	.139	.052
34	521	.992	.985	.091	.032
35	504	.992	.985	.080	.030
36	489	.992	.986	.084	.033
37	444	.986	.974	.107	.043
38	438	.992	.986	.089	.029
39	473	.998	.996	.046	.017
40	483	.989	.979	.096	.035
41	465	.992	.986	.078	.031
42	485	.986	.974	.125	.039
43	501	.992	.984	.091	.032

Table S15. Robust fit statistics for the second-order model across waves in the German repeated cross-sectional survey based on robust diagonally weighted least squares estimation.

Wave	n	CFI	TLI	RMSEA	SRMR
24	1,018	.998	.996	.053	.015
25	1,013	.991	.983	.108	.027
26	1,018	.994	.989	.091	.024
31	973	.991	.983	.094	.029
32	1,007	.992	.986	.095	.026
33	1,014	.988	.978	.105	.032
34	1,001	.994	.989	.082	.023
35	1,021	.990	.981	.100	.031
37	1,012	.988	.978	.098	.031
38	994	.992	.986	.088	.025
39	1,014	.997	.995	.049	.017
40	976	.995	.992	.069	.021
41	997	.993	.987	.078	.023
42	1,040	.985	.972	.113	.032
43	950	.995	.990	.078	.023
44	980	.994	.988	.076	.022
49	967	.991	.983	.102	.026
51	951	.995	.991	.075	.021

Table S16. Means, standard deviations, and Cronbach's α for the online experiment.

		Full sample			Control		Low pandemic fatigue		High pandemic fatigue	
Variable	Items	Mean	SD	α	Mean	SD	Mean	SD	Mean	SD
Cognitive risk perceptions	2	17.37	10.40	-	17.43	10.89	17.40	10.02	17.27	10.28
Pandemic fatigue	6	3.30	1.43	.85	3.29	1.45	3.08	1.36	3.55	1.43
Behavioral fatigue	3	2.94	1.48	.75	2.91	1.48	2.74	1.47	3.20	1.46
Information fatigue	3	3.66	1.73	.86	3.67	1.77	3.41	1.62	3.90	1.75
Physical distancing	1	5.96	1.48	-	5.99	1.46	6.07	1.47	5.81	1.51
Hygiene	1	6.08	1.30	-	6.14	1.29	6.11	1.34	5.98	1.26
Mask wearing	1	6.14	1.52	-	6.21	1.51	6.20	1.49	6.00	1.55
Information seeking	1	5.10	1.74	-	5.09	1.73	5.39	1.67	4.79	1.77
Behavioral intentions	4	5.82	1.15	.76	5.86	1.13	5.94	1.13	5.65	1.18

Note. Standard deviation (SD). Participants' cognitive risk perception was estimated by taking the product of one item measuring participants' assessment of how serious it would be for them to get infected and one item asking them to judge their own likelihood of contracting the virus.

Table S17. Item overview online experiment.

Variable	Item	Response format
Age	Please indicate your age	0-100 years
Gender	Please indicate your gender	(0) Male<br>(1) Female<br>(2) Other
Education	Please indicate your highest level of education	(1) Elementary school<br>(2) Secondary school<br>(3) High school<br>(4) Bachelor<br>(5) Master<br>(6) Ph.D.<br>(7) Other
Cognitive risk perceptions	On this page we kindly ask you to answer the following two questions concerning your perceived risk of contracting the novel coronavirus (COVID-19).	
	How likely do you think it is that you will be infected with the novel coronavirus (COVID-19)?	(1) Very unlikely<br>-<br>(7) Very likely
	How serious would it be for you if you contracted the novel coronavirus (COVID-19)?	(1) Not serious at all<br>-<br>(7) Very serious

Pandemic fatigue	On this page we kindly ask you to indicate how much you disagree or agree with the following statements.<br><br>I am tired of all the COVID-19 discussions in TV shows, newspapers, and radio programs, etc.<br><br>I am sick of hearing about COVID-19<br><br>When friends or family members talk about COVID-19, I try to change the subject because I do not want to talk about it anymore<br><br>I feel strained from following all of the behavioural regulations and recommendations around COVID-19<br><br>I am tired of restraining myself to save those who are most vulnerable to COVID-19<br><br>I am losing my spirit to fight against COVID-19	(1) Strongly disagree<br>-<br>(7) Strongly agree
Physical distancing	On this page we kindly ask you to indicate how much you disagree or agree with the following statements.<br><br>Over the next two weeks I will avoid physical contacts and keep a safe distance to people outside my own household	(1) Strongly disagree<br>-<br>(7) Strongly agree

Hygiene	On this page we kindly ask you to indicate how much you disagree or agree with the following statements.<br><br>Over the next two weeks I will wash my hands very often and thoroughly and/or use hand disinfectant frequently	(1) Strongly disagree<br>-<br>(7) Strongly agree
Mask wearing	On this page we kindly ask you to indicate how much you disagree or agree with the following statements.<br><br>Over the next two weeks I will wear a face mask whenever I am inside and cannot keep a safe physical distance to people outside my own household	(1) Strongly disagree<br>-<br>(7) Strongly agree
Information seeking	On this page we kindly ask you to indicate how much you disagree or agree with the following statements.<br><br>Over the next two weeks I will do everything I can to keep myself updated about the development of the pandemic, and stay informed about the current COVID-19 restrictions	(1) Strongly disagree<br>-<br>(7) Strongly agree

Table S18. Sociodemographics for the online experiment.

Variable	Control	Low pandemic fatigue	High Pandemic fatigue
**Age**			
Mean	36.37	35.48	34.83
SD	12.16	11.84	11.55
**Gender**			
Female	52.90%	47.17%	50.80%
Male	45.29%	51.13%	47.61%
Other	1.81%	1.70%	1.59%
**Education**			
Elementary school	0.00%	0.00%	0.00%
Secondary school	0.00%	0.57%	0.40%
High school	26.45%	24.91%	23.51%
Bachelor	45.29%	42.64%	44.82%
Master	18.30%	21.89%	20.92%
Ph.D.	3.44%	3.02%	3.78%
Other	6.52%	6.98%	6.57%

Note. Abbreviations: Standard deviation (SD).

Table S19. Very simple structure and Velicer's minimum average partial by number of factors based on polychoric correlations.

Factors	VVS1	VVS2	MAP
1	.92	.00	.04
2	.66	.75	.04
3	.39	.63	.06
4	.38	.49	.09
5	.28	.41	.14

Note. Very simple structure (VVS); minimum average partial (MAP).

Table S20. Standardized loadings, communalities, uniqueness, and complexity for the six items retained based on polychoric correlations.

Item	IF	BF	Communalities	Uniqueness	Complexity
1. I am tired of all the COVID-19 discussions in TV shows, newspapers, and radio programs, etc.	.88	-.05	.72	.28	1.00
2. I am sick of hearing about COVID-19.	.90	.02	.84	.16	1.00
3. When friends or family members talk about COVID-19, I try to change the subject because I do not want to talk about it anymore.	.53	.25	.52	.48	1.40
4. I feel strained from following all of the behavioral regulations and recommendations around COVID-19.	.03	.85	.75	.25	1.00
5. I am tired of restraining myself to save those who are most vulnerable to COVID-19.	.13	.62	.50	.50	1.10
6. I am losing my spirit to fight against COVID-19.	-.08	.78	.54	.46	1.00
Eigenvalues	1.99	1.88			
Proportion of variance	.33	.31			

Note. Response scale: 1 = strongly disagree, 2 = disagree, 3 = somewhat disagree, 4 = neutral/neither disagree nor agree, 5 = somewhat agree, 6 = agree, 7 = strongly agree. Abbreviations: Information fatigue (IF), behavioral fatigue (BF).

Supplementary References

1. Petherick, A. *et al.* A worldwide assessment of changes in adherence to COVID-19 protective behaviours and hypothesized pandemic fatigue. *Nat Hum Behav* **5**, 1145–1160 (2021).
2. Wright, L., Steptoe, A. & Fancourt, D. Trajectories of Compliance With COVID-19 Related Guidelines: Longitudinal Analyses of 50,000 UK Adults. *Annals of Behavioral Medicine* 2021.04.13.21255336 (2022) doi:10.1093/abm/kaac023.
3. MacIntyre, C. R. *et al.* Mask use, risk-mitigation behaviours and pandemic fatigue during the COVID-19 pandemic in five cities in Australia, the UK and USA: A cross-sectional survey. *International Journal of Infectious Diseases* **106**, 199–207 (2021).
4. Dolan, C. v. Factor analysis of variables with 2, 3, 5 and 7 response categories: A comparison of categorical variable estimators using simulated data. *British Journal of Mathematical and Statistical Psychology* **47**, 309–326 (1994).
5. Muthén, B. & Kaplan, D. A comparison of some methodologies for the factor analysis of non-normal Likert variables. *British Journal of Mathematical and Statistical Psychology* **38**, 171–189 (1985).
6. Flora, D. B., LaBrish, C. & Chalmers, R. P. Old and New Ideas for Data Screening and Assumption Testing for Exploratory and Confirmatory Factor Analysis. *Front Psychol* **3**, (2012).
7. Kaiser, H. F. A second generation little jiffy. *Psychometrika* **35**, 401–415 (1970).
8. Bartlett, M. S. Tests of significance in factor analysis. *British Journal of Psychology* **3**, 77–85 (1950).
9. Cattell, R. B. The scree test for the number of factors. *Multivariate Behav Res* **1**, 245–276 (1966).

10. Glorfeld, L. W. An Improvement on Horn's Parallel Analysis Methodology for Selecting the Correct Number of Factors to Retain. *Educ Psychol Meas* **55**, 377–393 (1995).

11. Horn, J. L. A rationale and test for the number of factors in factor analysis. *Psychometrika* **30**, 179–185 (1965).

12. Revelle, W. & Rocklin, T. Very simple structure: An alternative procedure for estimating the optimal number of interpretable factors. *Multivariate Behav Res* **14**, 403–414 (1979).

13. Velicer, W. F. Determining the number of components from the matrix of partial correlations. *Psychometrika* **41**, 321–327 (1976).

14. Watkins, M. W. *A Step-by-Step Guide to Exploratory Factor Analysis with R and RStudio*. (Routledge, 2020). doi:10.4324/9781003120001.

15. Finch, W. H. Using Fit Statistic Differences to Determine the Optimal Number of Factors to Retain in an Exploratory Factor Analysis. *Educ Psychol Meas* **80**, 217–241 (2020).

16. Clark, D. A. & Bowles, R. P. Model Fit and Item Factor Analysis: Overfactoring, Underfactoring, and a Program to Guide Interpretation. *Multivariate Behav Res* **53**, 544–558 (2018).

17. Li, C.-H. Confirmatory factor analysis with ordinal data: Comparing robust maximum likelihood and diagonally weighted least squares. *Behav Res Methods* **48**, 936–949 (2016).

18. Rosseel, Y. lavaan : An R Package for Structural Equation Modeling. *J Stat Softw* **48**, (2012).

19. Yoon, M. & Millsap, R. E. Detecting Violations of Factorial Invariance Using Data-Based Specification Searches: A Monte Carlo Study. *Struct Equ Modeling* **14**, 435–463 (2007).

20. Bowen, N. K. & Masa, R. D. Conducting Measurement Invariance Tests with Ordinal Data: A Guide for Social Work Researchers. *J Soc Social Work Res* **6**, 229–249 (2015).

21. Hoffman, L. *Longitudinal Analysis: Modeling Within-Person Fluctuation and Change*. (Routledge, 2015). doi:10.4324/9781315744094.

22. Curran, P. J. & Bauer, D. J. The Disaggregation of Within-Person and Between-Person Effects in Longitudinal Models of Change. *Annu Rev Psychol* **62**, 583–619 (2011).

23. Satorra, A. & Bentler, P. M. Corrections to test statistics and standard errors in covariance structure analysis. in *Latent variables analysis: Applications for developmental research.* 399–419 (Sage Publications, Inc, 1994).
