# Supplementary material for: Development and validation of the pandemic fatigue scale
Source: Nat Commun. 2023 Oct 10;14:6352. doi: 10.1038/s41467-023-42063-2 (PMC10564944; doi:10.1038/s41467-023-42063-2)
Supplement: Supplementary file 3 — Reporting Summary [file 41467_2023_42063_MOESM3_ESM.pdf]

nature portfolio

Corresponding author(s): Lau Lilleholt

Last updated by author(s): Jun 15, 2023

Reporting Summary

Nature Portfolio wishes to improve the reproducibility of the work that we publish. This form provides structure for consistency and transparency in reporting. For further information on Nature Portfolio policies, see our Editorial Policies and the Editorial Policy Checklist.

Statistics

For all statistical analyses, confirm that the following items are present in the figure legend, table legend, main text, or Methods section.

n/a	Confirmed	
☐	☒	The exact sample size (*n*) for each experimental group/condition, given as a discrete number and unit of measurement
☐	☒	A statement on whether measurements were taken from distinct samples or whether the same sample was measured repeatedly
☐	☒	The statistical test(s) used AND whether they are one- or two-sided<br>*Only common tests should be described solely by name; describe more complex techniques in the Methods section.*
☐	☒	A description of all covariates tested
☐	☒	A description of any assumptions or corrections, such as tests of normality and adjustment for multiple comparisons
☐	☒	A full description of the statistical parameters including central tendency (e.g. means) or other basic estimates (e.g. regression coefficient) AND variation (e.g. standard deviation) or associated estimates of uncertainty (e.g. confidence intervals)
☐	☒	For null hypothesis testing, the test statistic (e.g. *F*, *t*, *r*) with confidence intervals, effect sizes, degrees of freedom and *P* value noted<br>*Give P values as exact values whenever suitable.*
☒	☐	For Bayesian analysis, information on the choice of priors and Markov chain Monte Carlo settings
☐	☒	For hierarchical and complex designs, identification of the appropriate level for tests and full reporting of outcomes
☐	☒	Estimates of effect sizes (e.g. Cohen's *d*, Pearson's *r*), indicating how they were calculated

Our web collection on statistics for biologists contains articles on many of the points above.

Software and code

Policy information about availability of computer code

Data collection	All Danish data was collected using the survey software formr version v.020.6 (https://formr.org)<br><br>All German data was collected using the survey software from UNIPARK (https://www.unipark.com)
Data analysis	All analyses were conducted in R version 4.2.2. with the following packages:<br><br>Attached base packages:<br>stats<br>graphics<br>grDevices<br>utils<br>datasets<br>methods<br>base<br><br>Other attached packages:<br>report_0.5.7<br>multilevelTools_0.1.1<br>JWileymisc_1.4.0

April 2023

extrafont_0.19
PupillometryR_0.0.4
rlang_1.1.1
dplyr_1.1.2
ggpubr_0.6.0
reshape2_1.4.4
sjPlot_2.8.14
ggplot2_3.4.2
doBy_4.6.16
lme4_1.1-32
Matrix_1.5-4
jtools_2.2.1
corrplot_0.92
semTools_0.5-6
lavaan_0.6-15
semPlot_1.1.6
paran_1.5.2
MASS_7.3-58.3
psych_2.3.3
readr_2.1.4

Loaded via a namespace (and not attached):
backports_1.4.1
Hmisc_5.0-1
VGAM_1.1-8
systemfonts_1.0.4
plyr_1.8.8
igraph_1.4.2
splines_4.2.2
TH.data_1.1-2
digest_0.6.31
htmltools_0.5.5
lmerTest_3.1-3
fansi_1.0.4
magrittr_2.0.3
checkmate_2.1.0
lisrelToR_0.1.5
cluster_2.1.4
extraoperators_0.1.1
tzdb_0.3.0
openxlsx_4.2.5.2
modelr_0.1.11
RcppParallel_5.1.7
svglite_2.1.1
sandwich_3.0-2
extrafontdb_1.0
jpeg_0.1-10
sem_3.1-15
colorspace_2.1-0
rvest_1.0.3
xfun_0.38
crayon_1.5.2
microbenchmark_1.4.9 survival_3.5-5
zoo_1.8-12
glue_1.6.2
kableExtra_1.3.4
gtable_0.3.3
emmeans_1.8.5
webshot_0.5.4
MatrixModels_0.5-1
mi_1.1
sjstats_0.18.2
sjmisc_2.8.9
car_3.1-2
Rttf2pt1_1.3.12
rms_6.6-0
DEoptimR_1.0-12
SparseM_1.81

abind_1.4-5
scales_1.2.1
mvtnorm_1.1-3
rstatix_0.7.2
ggthemes_4.2.4
ggeffects_1.2.1
Rcpp_1.0.10
viridisLite_0.4.1
xtable_1.8-4
performance_0.10.3
htmlTable_2.4.1
foreign_0.8-84
Formula_1.2-5
stats4_4.2.2
httr_1.4.5
htmlwidgets_1.6.2
fstcore_0.9.14
mice_3.15.0
pkgconfig_2.0.3
XML_3.99-0.14
multcompView_0.1-9
nnet_7.3-18
kutils_1.70
utf8_1.2.3
tidyselect_1.2.0
munsell_0.5.0
tools_4.2.2
cli_3.6.1
generics_0.1.3
sjlabelled_1.2.0
broom_1.0.4
fdrtool_1.2.17
evaluate_0.20
stringr_1.5.0
fastmap_1.1.1
arm_1.13-1
knitr_1.42
robustbase_0.95-1
zip_2.3.0
pander_0.6.5
purrr_1.0.1
glasso_1.11
pbapply_1.7-0
nlme_3.1-162
quantreg_5.95
xml2_1.3.3
compiler_4.2.2
rstudioapi_0.14
png_0.1-8
ggsignif_0.6.4
tibble_3.2.1
pbivnorm_0.6.0
stringi_1.7.12
qgraph_1.9.4
rockchalk_1.8.157
lattice_0.21-8
nloptr_2.0.3
vctrs_0.6.2
pillar_1.9.0
lifecycle_1.0.3
OpenMx_2.21.8
estimability_1.4.1
cowplot_1.1.1
data.table_1.14.8
insight_0.19.1
corpcor_1.6.10
R6_2.5.1
gridExtra_2.3

codetools_0.2-19
polspline_1.1.22
boot_1.3-28.1
gtools_3.9.4
withr_2.5.0
mnormt_2.1.1
Deriv_4.1.3
multcomp_1.4-23
mgcv_1.8-42
bayestestR_0.13.1
parallel_4.2.2
hms_1.1.3
quadprog_1.5-8
fst_0.9.8
grid_4.2.2
rpart_4.1.19
tidyr_1.3.0
coda_0.19-4
minqa_1.2.5
rmarkdown_2.21
carData_3.0-5
numDeriv_2016.8-1.1
base64enc_0.1-3

For manuscripts utilizing custom algorithms or software that are central to the research but not yet described in published literature, software must be made available to editors and reviewers. We strongly encourage code deposition in a community repository (e.g. GitHub). See the Nature Portfolio guidelines for submitting code & software for further information.

Data

Policy information about availability of data

All manuscripts must include a data availability statement. This statement should provide the following information, where applicable:

- Accession codes, unique identifiers, or web links for publicly available datasets
- A description of any restrictions on data availability
- For clinical datasets or third party data, please ensure that the statement adheres to our policy

The data from the online experiment and the Danish and German repeated cross-sectional surveys used herein have been deposited on the Open Science Framework at: https://osf.io/xd463/ (DOI: 10.17605/OSF.IO/XD463). Please note that we—in line with the European General Data Protection Regulation—are unable to publicly share the raw data of the Danish panel survey because it contains personal identifiers that were linked to sensitive personal information (even though the data are stored in a (pseudo)anonymized format now). Instead, we provide an exemplary synthetic version of this data created with the synthpop package in R on the Open Science Framework : https://osf.io/xd463/ (DOI: 10.17605/OSF.IO/XD463). Raw data from the Danish panel survey is available upon request, but only after an appropriate data processing agreement can and has been signed. The data obtained from Our World in Data is available at: https://ourworldindata.org/coronavirus.

Research involving human participants, their data, or biological material

Policy information about studies with human participants or human data. See also policy information about sex, gender (identity/presentation), and sexual orientation and race, ethnicity and racism.

Reporting on sex and gender	Participants gender was assessed using self-report and considered in the analyses. The raw data including data on participants gender is available via: https://osf.io/xd463/?view_only=7dfd0586c05f450b8c4421cfc18c9d84
Reporting on race, ethnicity, or other socially relevant groupings	Not relevant
Population characteristics	The cross-sectional and panel surveys comprise of largely representative samples from Denmark and Germany. The experiment relies on U.S. participants from Prolific (https://www.prolific.co). The samples used have the following sample characteristics:<br><br>Danish Danish Repeated Cross-Sectional Survey: N = 15,985 (54.60% female, 45.18% male, 0.22% other; Mage = 56.54, SDage = 15.47 years)<br><br>German Repeated Cross-Sectional Survey: N = 17,946 (50.69% female, 49.31% male; Mage = 45.07, SDage = 15.72 years)<br><br>Danish Panel Survey: Sociodemographic information for all participants in each of the nine waves of the Danish panel survey is presented in Table S3<br><br>Experiment: N = 1,584 {50.32% female, 47.98% male, 1.70% other; Mage = 35.58, SDage = 11.87 years)

Recruitment

Danish Danish Repeated Cross-Sectional Survey:
In 2020, following data handling approval from the Faculty of Social Sciences of the University of Copenhagen (#514-0136/20-2000), the second author received contact information for two representative samples regarding age and gender of approximately 100,000 adult Danish citizens from Statistics Denmark (https://www.dst.dk/en). From these samples, random non-overlapping subsets of 5,250-8,500 Danes were invited via the official digital mail system in Denmark (https://www.e-boks.com/danmark/en) every other week from 2020-10-19 to 2021-09-20 to participate in the Danish repeated cross-sectional survey. The Danish repeated cross-sectional survey was set up and run in formr. Participation was voluntary, and informed consent was obtained from all participants. Participants where not compensated for their participation. All participants who experienced technical issues while filling out the survey were excluded from the final dataset. The general study protocol for the Danish repeated cross-sectional survey as well as the Danish panel survey (see below; https://www.psycharchives.org/en/item/8a92091d-a1b6-42ac-ae53-7ca70ed2ccc2) received ethical approval from the Institutional Review Board at the Copenhagen Center for Social Data Science, University of Copenhagen.

German Repeated Cross-Sectional Survey:
The study obtained ethical clearance from the University of Erfurt Internal Review Board (#20200302/20200501), and all participants provided informed consent prior to participation. The study involved a weekly to fortnightly repeated cross-sectional survey with approximately 1,000 non-overlapping individuals participating in each wave, using non-probability quota samples representative of the German population regarding age, gender, and federal state. The German repeated cross-sectional survey was set up and run using UNIPARK (https://www.unipark.com). Participants were compensated by the data collection company Respondi (https://www.respondi.com) for their participation. No participants were excluded from the final dataset.

Procedure Danish Panel Survey:
Via the same procedure as for the Danish repeated cross-sectional survey, the second author received contact information for a representative sample regarding age and gender of approximately 100,000 adult Danish citizens from Statistics Denmark in 2018. From this sample, a random subset of 15,000 Danes was invited to participate in the Danish panel survey via the official digital mail system in Denmark. Like the Danish repeated cross-sectional survey, the Danish panel survey was set up and run in formr. Participation was voluntary, and informed consent was obtained from all participants. Participants where compensated for their participation via a lottery in which they could win one of 30 vouchers worth 2,000 DKK (approximately US $305 at the time of the study) each.

Experiment:
We recruited a total of 1,854 U.S. participants via Prolific. Ethical clearance was obtained from the Institutional Review Board at the Department of Psychology, University of Copenhagen (#IP-IRB/22012021). All participants provided informed consent prior to participation. The experiment took approximately seven minutes to complete, and participants were paid a flat fee of £0.75 for their participation.

Problems of self-selection:
As with any other study relying on surveys and online experiments there is a risk that the samples obtained to some extent is affected by self-selection bias.

Ethics oversight

Danish Repeated Cross-Sectional and Panel Survey: The general study protocol for the Danish repeated cross-sectional survey as well as the Danish panel survey (https://www.psycharchives.org/en/item/8a92091d-a1b6-42ac-ae53-7ca70ed2ccc2) received ethical approval from the Institutional Review Board at the Copenhagen Center for Social Data Science, University of Copenhagen. We further obtained a data handling approval from the University of Copenhagen (#514-0136/20-2000)

German Repeated Cross-Sectional Survey: The study obtained ethical clearance from the University of Erfurt Internal Review Board (#20200302/20200501)

Experiment: Ethical clearance was obtained from the Institutional Review Board at the Department of Psychology, University of Copenhagen (#IP-IRB/22012021).

Note that full information on the approval of the study protocol must also be provided in the manuscript.

Field-specific reporting

Please select the one below that is the best fit for your research. If you are not sure, read the appropriate sections before making your selection.

☐ Life sciences ☒ Behavioural & social sciences ☐ Ecological, evolutionary & environmental sciences

For a reference copy of the document with all sections, see nature.com/documents/nr-reporting-summary-flat.pdf

Behavioural & social sciences study design

All studies must disclose on these points even when the disclosure is negative.

Study description

Cross-sectional surveys, longitudinal panel surveys, and randomized experiment.

Research sample

The cross-sectional and panel surveys comprise of largely representative samples from Denmark and Germany. The experiment relies on a convenience sample (i.e., non-representative) of U.S. participants from Prolific (https://www.prolific.co). The rationale for using

these samples is that we had easy access to them. The samples used have the following sample characteristics:

Danish Danish Repeated Cross-Sectional Survey: N = 15,985 (54.60% female, 45.18% male, 0.22% other; Mage = 56.54, SDage = 15.47 years)

German Repeated Cross-Sectional Survey: N = 17,946 (50.69% female, 49.31% male; Mage = 45.07, SDage = 15.72 years)

Danish Panel Survey: Sociodemographic information for all participants in each of the nine waves of the Danish panel survey is presented in Table S3

Experiment: N = 1,584 (50.32% female, 47.98% male, 1.70% other; Mage = 35.58, SDage = 11.87 years)

Sampling strategy

Danish Danish Repeated Cross-Sectional Survey:
In 2020, following data handling approval from the Faculty of Social Sciences of the University of Copenhagen (#514-0136/20-2000), the second author received contact information for two representative samples regarding age and gender of approximately 100,000 adult Danish citizens from Statistics Denmark (https://www.dst.dk/en). From these samples, random non-overlapping subsets of 5,250-8,500 Danes were invited via the official digital mail system in Denmark (https://www.e-boks.com/danmark/en) every other week from 2020-10-19 to 2021-09-20 to participate in the Danish repeated cross-sectional survey. The Danish repeated cross-sectional survey was set up and run in formr. Participation was voluntary, and informed consent was obtained from all participants. Participants where not compensated for their participation. All participants who experienced technical issues while filling out the survey were excluded from the final dataset. The general study protocol for the Danish repeated cross-sectional survey as well as the Danish panel survey (see below; https://www.psycharchives.org/en/item/8a92091d-a1b6-42ac-ae53-7ca70ed2ccc2) received ethical approval from the Institutional Review Board at the Copenhagen Center for Social Data Science, University of Copenhagen. No statistical method were used to predetermine the sample sizes for the Danish repeated cross-sectional surveys, but we aimed to recruit at least 500 participants per wave, allowing us to detect a correlation of 0.15 with power 1- B = .95 and a two-tailed alpha level of .05.

German Repeated Cross-Sectional Survey:
The study obtained ethical clearance from the University of Erfurt Internal Review Board (#20200302/20200501), and all participants provided informed consent prior to participation. The study involved a weekly to fortnightly repeated cross-sectional survey with approximately 1,000 non-overlapping individuals participating in each wave, using non-probability quota samples representative of the German population regarding age, gender, and federal state. The German repeated cross-sectional survey was set up and run using UNIPARK (https://www.unipark.com). Participants were compensated by the data collection company Respondi (https://www.respondi.com) for their participation. No participants were excluded from the final dataset. No statistical method were used to predetermine the sample sizes for the German cross-sectional surveys, but we aimed to recruit at least 1,000 participants per wave, allowing us to detect a correlation of 0.11 with power 1- B = .95 and a two-tailed alpha level of .05.

Procedure Danish Panel Survey:
Via the same procedure as for the Danish repeated cross-sectional survey, the second author received contact information for a representative sample regarding age and gender of approximately 100,000 adult Danish citizens from Statistics Denmark in 2018. From this sample, a random subset of 15,000 Danes was invited to participate in the Danish panel survey via the official digital mail system in Denmark. Like the Danish repeated cross-sectional survey, the Danish panel survey was set up and run in formr. Participation was voluntary, and informed consent was obtained from all participants. Participants where compensated for their participation via a lottery in which they could win one of 30 vouchers worth 2,000 DKK (approximately US $305 at the time of the study) each. No statistical method were used to predetermine the sample sizes for the Danish panel surveys, but we aimed to recruit at least 300 participants per wave, allowing us to detect a correlation of 0.20 with power 1- B = .95 and a two-tailed alpha level of .05.

Experiment:
We recruited a total of 1,854 U.S. participants via Prolific. Ethical clearance was obtained from the Department of Psychology, University of Copenhagen's IRB (# IP-IRB / 22012021). To determine an appropriate sample size for the experiment, we conducted an a priori power analysis based on results from a pilot study designed to test the experimental manipulation (n = 299) using G*Power. Aiming to be able to detect a small effect size (Cohen's d =.20) in an independent samples t-test with a two-tailed alpha level of .05 and high statistical power (1- B = .90), the a priori power analysis revealed that a total of 1,581 participants would be sufficient (i.e., 527 participants per condition). To compensate for potential exclusions, we decided to oversample by approximately 15% and thus aimed to recruit a total of 1,850 participants.

Data collection

Procedure Danish Repeated Cross-Sectional Survey:
In 2020, following data handling approval from the Faculty of Social Sciences of the University of Copenhagen (#514-0136/20-2000), the second author received contact information for two representative samples regarding age and gender of approximately 100,000 adult Danish citizens from Statistics Denmark (https://www.dst.dk/en). From these samples, random non-overlapping subsets of 5,250-8,500 Danes were invited via the official digital mail system in Denmark (https://www.e-boks.com/danmark/en) every other week from 2020-10-19 to 2021-09-20 to participate in the Danish repeated cross-sectional survey. The Danish repeated cross-sectional survey was set up and run in formr. Participation was voluntary, and informed consent was obtained from all participants. Participants where not compensated for their participation. All participants who experienced technical issues while filling out the survey were excluded from the final dataset. The general study protocol for the Danish repeated cross-sectional survey as well as the Danish panel survey (see below; https://www.psycharchives.org/en/item/8a92091d-a1b6-42ac-ae53-7ca70ed2ccc2) received ethical approval from the Institutional Review Board at the Copenhagen Center for Social Data Science, University of Copenhagen. A total of 15,985 respondents participated in the 25 waves of the Danish repeated cross-sectional survey considered herein without experiencing any technical issues (54.60% female, 45.18% male, 0.22% other; Mage= 56.54, SDage = 15.47 years). Sociodemographic information for all participants in the Danish repeated cross-sectional survey is presented in Table S1. The response and completion rate for each wave of the Danish repeated cross-sectional survey considered herein is presented in Table S2. Across the 25 waves of the Danish repeated cross-sectional survey used for this investigation, some variables were assessed consistently, while others were only measured sporadically. Links to an overview of all variables assessed in the 25 waves of the Danish repeated cross-sectional

survey can be found at: https://osf.io/xd463/

Procedure German Repeated Cross-Sectional Survey
The study obtained ethical clearance from the University of Erfurt Internal Review Board (#20200302/20200501), and all participants provided informed consent prior to participation. The study involved a weekly to fortnightly repeated cross-sectional survey with approximately 1,000 non-overlapping individuals participating in each wave, using non-probability quota samples representative of the German population regarding age, gender, and federal state. The German repeated cross-sectional survey was set up and run using UNIPARK (https://www.unipark.com). Participants were compensated by the data collection company Respondi (https://www.respondi.com) for their participation. No participants were excluded from the final dataset. A total of 17,946 respondents participated in the 18 waves of the German repeated cross-sectional survey considered herein (50.69% female, 49.31% male; Mage= 45.07, SDage = 15.72 years). The 18 waves of the German repeated cross-sectional survey used for this investigation were collected between 2020-10-27 and 2021-09-07. Sociodemographic information for all participants in the German repeated cross-sectional survey is presented in Table S1. As in the Danish repeated cross-sectional survey, some variables of the German repeated cross-sectional survey were measured consistently across all waves, while others were only assessed sporadically. An overview of all variables measured in the 18 waves of the German repeated cross-sectional survey can be found at: http://dx.doi.org/10.23668/psycharchives.2776.

Procedure Danish Panel Survey
Via the same procedure as for the Danish repeated cross-sectional survey, the second author received contact information for a representative sample regarding age and gender of approximately 100,000 adult Danish citizens from Statistics Denmark in 2018. From this sample, a random subset of 15,000 Danes was invited to participate in the Danish panel survey via the official digital mail system in Denmark. Like the Danish repeated cross-sectional survey, the Danish panel survey was set up and run in formr. Participation was voluntary, and informed consent was obtained from all participants. Participants where compensated for their participation via a lottery in which they could win one of 30 vouchers worth 2,000 DKK (approximately US $305 at the time of the study) each. A total of 2,546 respondents participated in the first wave of the Danish panel survey and were thus invited to participate in the subsequent waves of the survey. Herein, we use data from waves 11-19 of the Danish panel survey which was collected between 2020-10-19 and 2021-06-21. Across these nine waves, between 341 and 438 respondents participated in each wave. All observations in which participants experienced technical issues while filling out the survey were excluded from the final dataset. Sociodemographic information for all participants in each of the nine waves of the Danish panel survey is presented in Table S3. As in both the Danish and German repeated cross-sectional surveys, some variables of the Danish panel survey were measured consistently across all waves, while others were only assessed sporadically. Links to an overview of all variables measured in the nine waves of the Danish panel survey can be found at: https://osf.io/xd463/

Scales and Measures:
To best capture people’s perceptions, emotions, and behavioral reactions to the COVID-19 pandemic, all COSMO surveys were specifically tailored to each country. Although there is a substantial overlap between the COSMO surveys conducted in Denmark and Germany, there are also some differences with regard to the content of the surveys as well as how certain variables were assessed. Across both countries, participants’ cognitive and affective risk perceptions regarding COVID-19, their experiences of pandemic fatigue, and their chronic disease status were measured in the exact same manner. Participants’ worries about potential personal and societal consequences of the pandemic, level of institutional trust, physical distancing, hygienic practices, mask wearing, information seeking, age, gender, education, and employment status (repeated cross-sectional surveys only) were also measured in both Denmark and Germany but with slightly different items and/or response formats. Finally, respondent’s feelings of optimism about the future, negative affect, and empathy towards those most vulnerable to COVID-19, as well as their personality characteristics in terms of the HEXACO dimensions were only assessed in Denmark. All variables, with the exception of sociodemographics (i.e., age, gender, education, employment, and chronic disease status), were measured with either a five- or seven-point Likert-type scale with different anchors. In both the Danish and German surveys, participants had the opportunity to answer ‘Not relevant’ or ‘Don’t know’ to some items. In all cases, except for chronic disease status, we treated these responses as missing. Mean scores, standard deviations, and Cronbach’s α for all scales considered herein can be found in Tables S4 – S6. In Tables S7 – S9 we further provide an overview of all scales and items from the Danish and German repeated cross-sectional survey, as well as the Danish panel survey used in this investigation.

Procedure Online Experiment:
The online experiment was preregistered via aspredidcted.org on 2021-01-28 (see https://aspredicted.org/ua3ca.pdf) and set up and run in formr (https://formr.org). All confirmatory analyses correspond to the preregistered analysis plan. Ethical clearance was obtained from the Institutional Review Board at the Department of Psychology, University of Copenhagen (#IP-IRB/22012021). All participants provided informed consent prior to participation. The experiment took approximately seven minutes to complete, and participants were paid a flat fee of £0.75 for their participation. In the first part of the experiment, all participants were asked to provide information about their age, gender, and education, as well as to respond to two items assessing their cognitive risk perceptions regarding COVID-19 (i.e., “How likely do you think it is that you will be infected with the novel coronavirus (COVID-19)?” and “How serious would it be for you if you contracted the novel coronavirus (COVID-19)?”). Next, they were all randomized into one of three conditions—control, low, and high pandemic fatigue—and asked to complete a brief self-reflection task designed to manipulate their experience of pandemic fatigue (see Wildschut et al., 2006, for a similar self-reflection task) by specifically targeting the (de)motivational aspect of pandemic fatigue (i.e., feeling demotivated towards following recommended health-protective behaviors, including keeping oneself informed about the pandemic). In particular, participants in the low/high pandemic fatigue condition were presented with the following instruction: “Using the space provided below, please spend the next few minutes to describe some of the things that, over the last two weeks, have motivated/demotivated you to follow recommended protective behaviors (e.g., physical distancing, mask wearing, hygienic practices) and keep yourself informed about the COVID-19 pandemic”. In contrast, participants in the Control condition were given the following instruction: “Using the space provided below, please spend the next few minutes to describe some of the ordinary things that have happened over the last two weeks and affected your behavior in some way”.

The decision to focus on the (de)motivational aspect of pandemic fatigue was made on the basis of both methodological and theoretical considerations. First and foremost, we decided to focus on the (de)motivational aspect of pandemic fatigue because it allowed us to straightforwardly manipulate the experience of pandemic fatigue in opposite directions by simply asking participants to

reflect upon what motivated/demotivated them to adhere to recommended health-protective behaviors in the past few weeks. Second, we focused on this aspect because the feeling of weariness and exhaustion that also characterizes pandemic fatigue arguably is more perennial in nature and thus less susceptible to undergo rapid changes in response to time varying situational factors, including that of simple experimental manipulations.

Finally, after completing the brief self-reflection task, all participants were asked to complete the PFS and to respond to four items assessing their intention to adhere to recommendations regarding physical distancing (i.e., "Over the next two weeks I will avoid physical contacts and keep a safe distance to people outside my own household"), hygienic practices ("Over the next two weeks I will wash my hands very often and thoroughly and/or use hand disinfectant frequently"), and mask wearing ("Over the next two weeks I will wear a face mask whenever I am inside and cannot keep a safe physical distance to people outside my own household"), as well as to keep themselves informed about the pandemic and current COVID-19 restrictions ("Over the next two weeks I will do everything I can to keep myself updated about the development of the pandemic, and stay informed about the current COVID-19 restrictions"). Both the PFS and the four items assessing participants' intentions to adhere to recommendations regarding physical distancing, hygienic practices, and mask wearing as well as to keep themselves informed about the pandemic and current COVID-19 restrictions were answered on a 7-point Likert scale ranging from 1 = "Strongly disagree" to 7 = "Strongly agree". Mean scores, standard deviations, and Cronbach's α for all measures obtained in the experiment are presented in Table S16. An overview of all items and scales used in the experiment is available in Table S17.

Timing

The Danish cross-sectional data was collected between: 2020-10-19 to 2021-09-20.

The German cross-sectional data was collected between: 2020-10-27 and 2021-09-07

The Danish Panel data was collected between: 2020-10-19 and 2021-06-21

The experimental study was conducted on: 2021-01-28

Data exclusions

All participants who experienced technical issues while filling out the Danish repeated cross-sectional survey were excluded from the final dataset. Similarly all observations in which participants experienced technical issues while filling out the Danish panel survey were excluded from the final dataset.

In the German repeated cross-sectional data no participants were excluded from the final dataset.

Participants In both the Danish and German surveys, participants had the opportunity to answer 'Not relevant' or 'Don't know' to some items. In all cases, except for chronic disease status, we treated these responses as missing. The number of excluded observations differ based on the specific model in question. The exact number of observations used for each analysis is described in the manuscript.

In the experiment we excluded all participants who wrote fewer than 100 characters (including spaces) in the self-reflection task (n = 245) or who failed at least one of the two attention checks (n = 10). Moreover, we also excluded participants who experienced technical issues during the experiment (n =15).

Non-participation

The number of eligible participants who participated in each wave of the Danish cross-sectional and panel survey can be found in Table S2 and S3, respectively. For the German cross-sectional survey, we are uable to include this information, because this survey was collected by an external panel provider (ie., Respondi;https://www.respondi.com/EN/) who only provided us with complete responses (i.e., participants who completed the survey in its entirety). The reasons for non-participation in the Danish cross-sectional and panel survey are unknown.

A total of 2,075 participants entered the online experiment but only 1,854 completed it. The reasons for non-completion are unknown.

Randomization

Participants in the experimental study was randomly allocated to one of the following three experimental conditions: control, low pandemic fatigue, and high pandemic fatigue.

No randomization was carried out in the Danish and German repeated cross-sectional surveys, nor in the Danish panel survey, and we do thus not draw any causal conclusion on the basis of these data.

Reporting for specific materials, systems and methods

We require information from authors about some types of materials, experimental systems and methods used in many studies. Here, indicate whether each material, system or method listed is relevant to your study. If you are not sure if a list item applies to your research, read the appropriate section before selecting a response.

Materials & experimental systems

n/a	Involved in the study
☒	☐ Antibodies
☒	☐ Eukaryotic cell lines
☒	☐ Palaeontology and archaeology
☒	☐ Animals and other organisms
☒	☐ Clinical data
☒	☐ Dual use research of concern
☒	☐ Plants

Methods

n/a	Involved in the study
☒	☐ ChIP-seq
☒	☐ Flow cytometry
☒	☐ MRI-based neuroimaging
